# Precipitation timing mediates life-stage and population-level associations with climate for an indicator species

**DOI:** 10.1038/s41598-025-20796-y

**Published:** 2025-10-23

**Authors:** Shawn T. O’Neil, Carl G. Lundblad, Brianne E. Brussee, John C. Tull, Michael L. Casazza, Justin R. Small, Cameron L. Aldridge, Peter S. Coates

**Affiliations:** 1 U.S. Geological Survey, Western Ecological Research Center, Dixon, CA USA; 2 U.S. Geological Survey, Western Ecological Research Center, Reno, NV USA; 3 U.S. Geological Survey, Southwest Climate Adaptation Science Center, Reno, NV USA; 4 Nevada Department of Wildlife, Reno, NV USA; 5 U.S. Geological Survey, Fort Collins Science Center, Fort Collins, CO USA

**Keywords:** Climate effects, Biodiversity, Drought, Habitat loss, Hierarchical model, Sagebrush conservation, Bioinformatics, Biogeochemistry, Climate-change ecology, Ecosystem ecology, Population dynamics, Ecological modelling

## Abstract

Global climate change is contributing to declines in biodiversity, although changes vary across geographic regions and species. The iconic greater sage-grouse (*Centrocercus urophasianus;* sage-grouse) is central to conservation within the North American sagebrush ecosystem, yet its vulnerability to climate effects remains poorly understood. We used hierarchical models to explore weather and climatic influences on sage-grouse across nearly four decades, focusing first on pattern (population change), then process (demographic life stage). We quantified eight indicators of temperature and/or moisture conditions across time lags of 0–1.5 years leading up to current year breeding activities. Sage-grouse population growth tended to increase following above-average growing season precipitation. Precipitation or drought timing influenced whether process effects were positive or negative. More summer and/or fall moisture corresponded with increases in subsequent year brood and adult survival, while concurrent seasonal drought led to reductions in nest survival. Positive concurrent spring drought associations with adult and brood survival were largely outweighed by positive prior year moisture effects. Density-dependence and indirect effects of precipitation through vegetation and forage resource pathways likely contributed to nuanced responses across life stages. Our research improves mechanistic understanding of this indicator species’ sensitivity to climatic factors, while reinforcing the importance of large-scale conservation initiatives for sagebrush ecosystems.

**Supplementary Information:**

The online version contains supplementary material available at 10.1038/s41598-025-20796-y.

## Introduction

 Rapid losses in biodiversity are an anticipated consequence of global change^1,2^. Yet, changes in biodiversity are expected to vary geographically and across taxonomic groups, with declines occurring in some regions while increases occur in others (e.g., redistribution, range contraction, expansion)^3^. A key priority of wildlife conservation is to assess the relative climate vulnerabilities of species as a function of their life history traits, habitat requirements, dispersal abilities, and other aspects of their individual ecology^4–6^. For example, species already in decline may be vulnerable to additive or multiplicative effects of a warming climate, which interact with and exacerbate other threats to long-term population viability. Therefore, disentangling the influences of climatic factors from other environmental risk factors can guide conservation action and inform effective use of limited financial resources.

The greater sage-grouse (*Centrocercus urophasianus*; hereafter sage-grouse) is an iconic gallinaceous bird inhabiting sagebrush-dominant (*Artemisia* spp.) rangelands in the interior western United States and Canada. Sage-grouse are considered a “landscape species” due to their complex seasonal movements and requirements for large tracts of intact and heterogeneous sagebrush-dominant habitats^7^. Sage-grouse face a suite of threats that contribute to degraded sagebrush ecosystems across its range^8–10^. Consequently, sage-grouse have been petitioned for listing under the Endangered Species Act on multiple occasions^11^ and remain a species of conservation concern that influences conservation strategies and land use policy in the interior western United States^12,13^. Declining population trends in sage-grouse^14^ are attributed to fragmentation of the sagebrush biome through land conversion and infrastructure development, expansion of conifers, and invasion by exotic annual grasses and subsequent grass-wildfire feedback cycles^15–18^. Disturbances to sagebrush ecosystems can interact with changing climate conditions (e.g., annual grass invasion, grass-fire cycles) or operate independently (e.g., anthropogenic development). Such disturbances may be aggravated by future climate regimes, imposing additional stressors on already degraded habitats, and leading to further population decline.

Previous studies have demonstrated that variation in sage-grouse population abundance tracks climate variability. Metrics of population size and performance have been positively linked to precipitation and negatively associated with drought conditions^14,19–21^. Such effects are generally interpreted as responses to herbaceous vegetation dynamics that mediate the availability of food and cover resources^22^. Resulting interannual climate variability and its interaction with density dependent processes are thought to underlie sage-grouse population cycling and form the basis of sage-grouse life-history evolution^22–24^. However, other studies have reported negative effects of precipitation on sage-grouse vital rates presumably mediated by limits to thermoregulatory capacity, especially among non-adult life stages^25–28^. Considerable uncertainties remain about the mechanisms underlying relationships between sage-grouse population abundance and weather patterns, as well as the multitude of direct and indirect effects by which weather (short-term) and climatic (long-term) factors affect specific demographic rates.

Drought severity and frequency in the western United States are expected to increase, and wet-dry cycles may be amplified^29^ with accelerating climate change^30,31^. Climate-induced habitat changes, such as the spread of exotic annual grasses and concurrent changes to fire frequency, could further exacerbate sage-grouse population sensitivity to such variation^18,19,32–34^. Changes in type, timing, and frequency of extreme precipitation events may also occur under future climates. Although higher amounts of precipitation are generally understood to have net benefits to sage-grouse populations over coarse time scales^14,18–20^, the effects of extreme precipitation events and their timing are less understood^35,36^. Changes in precipitation type (e.g., reductions in snowpack) could reduce the availability of limited mesic resources that characterize brood-rearing habitat^37^ and which are associated with sage-grouse population density and recruitment^38–40^. Finally, directional change in global mean temperature and thermal extremes may impose additional direct and acute effects on sage-grouse through various life-stages^41^.

To address knowledge gaps and provide a baseline framework for understanding potential future climate impacts, we sought to understand the relative influences of precipitation, temperature, and drought on both pattern and process of sage-grouse populations inhabiting the Great Basin region of the USA. Sage-grouse populations in this region may be more sensitive to direct and indirect effects of climate change than elsewhere in their distribution, due to moisture limitation, extreme drought, and altered wildfire cycles^14,18^. To investigate pattern, we analyzed spatiotemporal contributions of precipitation and drought to inter-annual population growth rates from annual breeding ground (lek) data, considering multiple time lags and metrics spanning previous years while accounting for density dependence and other relevant environmental effects. We then explored processes potentially contributing to observed population pattern, wherein we focused on responses of sage-grouse to precipitation, temperature and drought during and leading up to key reproductive life history stages, including nest, brood, and adult survival. Although our analyses were conducted with observational data, and were thus largely exploratory, we found it useful to frame our analyses and interpretation of results in the context of two general guiding hypotheses: (1) life-stage specific survival is limited by climate-mediated effects on soil moisture conditions and resulting herbaceous vegetation dynamics that support the availability of protective cover and food resources, and (2) life-stage specific survival is limited by direct physiological exposure effects imposed by extreme wet, cold, and/or hot conditions. The first hypothesis assumes survival is positively associated with soil moisture availability that favors the growth of herbaceous vegetation associated with food (generally forbs and associated arthropod communities) and protective cover for multiple sage-grouse life stages^42–44^. Therefore, the first hypothesis predicts that survival will be positively associated with *lagged or cumulative* effects reflected in higher precipitation, lower aridity, or lower temperature that lead to higher sustained soil moisture availability. The second hypothesis assumes that survival could be directly limited by extreme weather conditions that exceed the thermoregulatory capacity and physiological limits of sage-grouse, especially among more vulnerable life stages (nests and broods)^45–47^. Therefore, the second hypothesis predicts that survival could be negatively associated with greater *concurrent* precipitation, extreme cold, or extreme heat. Inferences about the population-level pattern then follow from resulting insights and previous findings regarding life stage contributions to overall population rates of change^23,24^. Collectively, understanding spatiotemporal influences of temperature, precipitation, and cumulative drought on sage-grouse provides baseline information used to project population change and inform management under various future climate scenarios.

## Methods

### Data collection

Our study encompassed the Great Basin region of the USA, with demographic data collected at sites in California, Idaho, and Nevada, while lek count data were gathered from sage-grouse occupied range within California, Idaho, Nevada, and Oregon (Appendix S1-Fig. S1). A detailed study area description is available in Appendix S1.

### Sage-grouse lek data

State, federal, private, and non-profit agency personnel counted sage-grouse at leks each year, 1985–2021. Observers followed standardized field protocols^13,14,48^. Lek surveys occurred each spring, overlapping peak lek attendance (1 Mar – 30 May)^48,49^. Leks were often surveyed multiple times per season, and sometimes multiple times per day. However, the number of surveys per season varied widely over time (e.g., range of 2–77 total surveys per lek, for those receiving multiple counts) and often depended on the agency collecting the data^48^. We used maximum male counts for each lek and year. Maximum counts are commonly used to index population abundance and are assumed to be unbiased provided no long-term trend in detection probability occurs^50,51^. We used a standardized lek count database containing common attributes, criteria for inclusion, and consistent handling and filtering protocols^48^. Detailed description of the protocols used are available in O’Donnell et al.^48,52^. In addition, we filtered out leks with excessively sparse survey data (Appendix S2).

### Sage-grouse demographic data

We used standard telemetry methods to gather data on sage-grouse nest, brood, and adult survival. We used spotlighting^53,54^ to capture sage-grouse and fit them with very high frequency (VHF) transmitters^55^ (Advanced Telemetry Systems, Isanti, Minnesota) or GPS Platform Transmitter Terminals (GeoTrak, Inc., Apex, North Carolina) at 25 field sites (Appendix S1-Fig. S1) during spring and fall, 2003–2021 (Appendix S3). Sage-grouse ages were determined (adult: > 2 years old; yearling: after hatch year and < 2 years old), and nest locations, nest fate, and subsequent brood locations and fates were recorded from field observations throughout the nesting and brood-rearing seasons (details in Appendix S3).

### Statistical analysis

#### Overview

We investigated sage-grouse responses to inter-annual variability in climate and weather variables using four distinct data types (annual lek counts, nest, brood, and adult encounter histories generated from telemetry data) and corresponding hierarchical modeling frameworks. First, we used lek count data to model and estimate climatic and land cover influences on sage-grouse apparent population growth. Models of lek count data assume that changes in counts accurately reflect fluctuations in population abundance, provided inter-annual detection and attendance rates are approximately constant over multi-year time series^49,51,56^. The lek count model can infer habitat and climate effects on inter-annual population rate of change without assumptions about specific life stage contributions, thereby elucidating broad population-level patterns that correspond with environmental and climatic variation. Second, we modeled daily survival during three key life stages for sage-grouse: nest, brood, and adult/yearling during the overall reproductive period. We considered the Mar–Aug reproductive time period, coinciding with on-the-ground data collection. For survival analyses, we used binomial logistic exposure models fit to daily timestep encounter histories, based on observations from VHF or GPS telemetry-marked individuals that inferred active (nest active, individual or brood alive) vs. failed (i.e., nest failure, individual or brood mortality) status.

### Environmental covariates

For all models, we used fractional remotely sensed land cover data and weather and climate indices to capture dynamic and relevant environmental conditions, such as vegetation cover components, topography, mesic areas, anthropogenic development, and drought or precipitation patterns. Vegetation components included annual percent cover of shrub, sagebrush, non-sagebrush shrub, perennial forb and grass, annual forb and grass, and bare ground^57–59^. For topography, we included elevation, topographic roughness, topographic position, heat load index, and transformed aspect. Mesic areas included seasonal wetlands, mesic rangelands, wet meadows, and riparian areas^60^. Anthropogenic development was represented by percent developed imperviousness^61^, and patterns in drought and seasonal moisture were captured by spatiotemporal variation in precipitation (PPT), standardized precipitation index (SPI), standardized precipitation evapotranspiration index (SPEI), potential water deficit (PWD), vapor pressure deficit (VPD), winter and spring snow water equivalent (SWE), and temperature (daily minimums and maximums; TMIN, TMAX, respectively)^62,63^. We considered several different indices of precipitation and moisture availability because they differentially reflect hydrologic conditions. PPT reflects absolute cumulative precipitation, while SPI reflects cumulative precipitation standardized to local long-term average (i.e., 30-year normal). SPEI integrates precipitation and potential evapotranspiration and is standardized to local long-term average. VPD and PWD are indices of atmospheric aridity; VPD describes the difference between observed relative humidity and the temperature-specific atmospheric moisture capacity (the saturation point), while PWD describes the atmospheric water deficit relative to the demands of plant growth. Therefore, PPT and SPI are direct metrics of precipitation, that might better reflect the potential for certain exposure effects, while SPEI, VPD, and PWD are expected to better reflect the cumulative influences of precipitation and aridity on soil moisture conditions that drive herbaceous plant growth. Finally, SWE reflects moisture stored as snowpack and that might recharge soil moisture weeks or months after falling. For each covariate, we assumed scale-dependent responses^64^, and specified separate sets of candidate spatial scales for population (radial buffer distances of *r =* 2.5, 5, and 10 km) and life stage (*r* = 75, 167, 260, 370, 439, and 1,451 m) analyses, based on generalized movement distances of sage-grouse and their distributions relative to leks (Appendix S4). We assumed each life stage would be influenced at relatively local scales (and considered buffer distances derived from our telemetry datasets), whereas population growth rates would reflect the cumulative influence of each demographic process, more widely dispersed around the lek site (Appendix S4). At each spatial scale, we characterized local conditions using circular moving windows and calculated an exponential decay function, $\:\text{e}\text{x}\text{p}($-$\:d/\alpha\:)$, to represent proximity to features. Grid cell sizes for drought and precipitation (4 km) were coarser than those of other environmental covariates, so these were not scale-dependent. Descriptions, sources, and general hypotheses of potential effects for all covariates and scales are in Appendix S4.

We sought to identify appropriate time lags for all drought and precipitation indices. For population analyses, we posited that prior year drought and precipitation (PPT, SPI, SPEI, PWD, VPD) would affect current year abundance at leks, with their relative influences depending on conditions experienced during specific seasons, life stages, or cumulatively over the prior year (see Appendix S4-Figure S1). Hence, we considered averages across the previous calendar year (14 Mar–14 Mar), northern hemisphere water year (1 Oct–30 Sep; hereafter ‘water year’), northern hemisphere growing season (1 Apr–31 Oct; hereafter ‘growing season’), spring (1 Mar–31 May), summer (1 Jun–31 Aug), fall (1 Sep–30 Nov), and winter (1 Dec–28 Feb). Northern hemisphere water year is intended to capture a full annual precipitation cycle, beginning in the fall and coinciding with the accumulation of snowpack that may recharge soil moisture during the following spring and summer^65^. The northern hemisphere growing season is intended to capture the primary annual period of plant growth and development^66^.

To investigate process, we posited that individual sage-grouse, and their nests and broods, could be sensitive to negative, short-term effects of weather events such as heat waves, very cold temperatures, snow, and rain. Alternatively, more indirect carryover (lagged) effects from weather during previous seasons could influence current vegetation productivity, wherein more precipitation and less drought might have positive effects, and vice versa. As such, we considered current and previous month and overall spring (Mar–May) averages for all precipitation and temperature indices (PPT, SPI, SPEI, PWD, VPD, TMIN, TMAX). We also considered monthly minimums and maximums for TMIN and TMAX. To account for spring snowpack or late snow events, we included average and maximum spring SWE. For carryover effects, we considered averages spanning the previous calendar year, growing season, fall, and winter.

For all analyses, lek counts and sage-grouse locations were aligned spatially and temporally with underlying raster data using package *terra*^67^ in R 4.1.3^68^. All environmental covariates were standardized to their z-scores.

### Population state-space model

We used a hierarchical population state-space model (SSM)^69,70^ in a Bayesian framework to evaluate covariate influences on annual population rate of change (*λ*) from lek count data, 1985–2021. We modeled the maximum number of males (*N*) per lek (*i*) and year (*t*), expressed as

1 $$\begin{gathered} \log ({N_{i,t+1}})=\log ({N_{i,t}})+{r_{i,t}} \hfill \\ {r_{i,t}}\sim Normal({\mu _{{r_{i,t}}}},\sigma _{{{r_i}}}^{2}) \hfill \\ \end{gathered}$$

where *r*_*i, t*_ represented inter-annual intrinsic growth rate for each lek and year, $\:{\sigma\:}_{{r}_{i}}^{2}$ was process error per lek, and *N*_*i, t*_ were latent apparent abundances. We specified observation error as,

2 $${y_{i,t}}\sim Poisson({N_{i,t}})$$

where the maximum count (i.e., the observations), *y*_*i, t*_, represented a random draw from the latent apparent abundance at each lek-year. We estimated effects on the lek-level population trends by fitting the mean growth rate, $\:{\mu\:}_{{r}_{i,t}}$, as a function of covariates (Appendix S4),

3 $${\mu _{{r_{i,t}}}}={\alpha _R}+X_{{i,t}}^{\prime }{\beta _R}+{\theta _R}$$

where *α* was a random intercept for region (*R*), $\:{\text{X}}_{{{\text{i,t}}}}^{{\prime \:}} \beta _{{\text{R}}}$ represented environmental covariates at each lek location and year multiplied by region-specific coefficients, with a region-specific Gompertz density-dependence term, $\:{\theta\:}_{\text{R}}$, capturing prior year density influences (*N*_*i, t−1*_) on current year growth rates. Regions were determined from nested hierarchical population units representing sage-grouse sub-population structure^71^. From these units, we used a scale (level-5 polygons)^71^ large enough to capture multiple leks per unit after data filtering, while also representing areas used by interconnected sub-populations^72^ (Appendix S1). Hence, we specified regionally-varying population dynamics, with prior distributions expressed as:

4 $${\beta _{k,R}}\sim Normal({\mu _{{\beta _k}}},\sigma _{{{\beta _k}}}^{2})$$

5 $${\alpha _R}\sim Normal(\mu ,{\upsigma ^2})$$

We used 2-stage Bayesian variable selection to identify influential covariates from groups of correlated covariates (Appendix S5). Although this model formulation estimates region-specific effects, we focused our reporting on the broader population-level inference (i.e., ‘grand means’), as exploration at finer scales was considered beyond our current scope of investigation. Thus, region-specific effects were included to appropriately capture variance across a large study region with spatially unbalanced sample sizes, as the number of sampled leks was not equal among sampling units.

### Nest survival

We used a hierarchical logistic exposure model^73,74^ in a Bayesian framework to analyze nest survival. Daily encounter histories were developed to capture the interval between the first observation of the active nest, the last active observation, and the time of fate. Our model took the form:

6 $$ \begin{aligned} & \eta_{i} = \beta_{0} + \beta_{1} x_{1} + \beta_{2} x_{2} + \ldots + \beta_{k} x_{k} \\& DSR_{i} = exp(\eta_{i}) / (1+ exp(\eta_{i})) \\& S_{i} = DSR^{t}_{i} \\& y_{i} \sim Bernoulli(S_{i})\end{aligned}$$

where *η*_*i*_ represented the model intercept (*β*_*0*_) plus environmental covariates (*x*_*1*_, *x*_*2*_, *…*,* x*_*k*_) multiplied by coefficients (*β*_*1*,_ *β*_*2, …*,_ *β*_*k*_), indexed by nest (*i*). The daily survival rate (*DSR*) was modeled through the logit link function. Nest observations *y*_*i*_ were determined either successful (*y = 1*) or not (*y = 0*) over the associated exposure interval (time in days, *t*), with survival probability *S*_*i*_. We considered candidate environmental covariates plus age of female (adult vs. yearling), and nest initiation date to account for individual and seasonal variation. Covariates were grouped based on their correlation structure (Appendix S5), and we tested interactions between nest initiation and precipitation or drought covariates, to account for seasonal effects (e.g., temperature increases with later date) on survival responses. Bayesian variable and scale selection methods (Appendix S5) were used to identify an appropriate representation for the most influential covariates.

### Brood survival

Our model for brood survival followed the same structure as the nest survival model (Eq. 6) with some important distinctions. First, encounter histories were specified for each brood such that each observed location and date were included, and the number of days between locations were the exposure intervals (e.g., multiple intervals for each brood). As such, the underlying environmental covariates were aligned so that conditions experienced by the brood represented the interval preceding each observed location^75^. Brood intervals were recorded successful (*y = 1*) if ≥ 1 chick survived and failed (*y = 0*) otherwise. We assessed evidence for candidate environmental covariates (Appendix S5) plus covariates for hen age, brood age (days since hatch), and interactions between brood age and day of season. We tested interactions between environmental covariates and brood age, thus allowing effects to vary as broods aged and migrated to late brood-rearing habitats^75,76^. We also tested interactions between day of year and precipitation/drought covariates and used Bayesian variable selection (Appendix S5) to identify appropriate representations for the most influential covariates.

### Adult and yearling survival

We modeled adult and yearling survival similarly to brood survival, with exposure intervals representing number of days between observed locations, and environmental covariates aligned so that conditions experienced by individuals represented the most recent interval preceding each location. Intervals were indicated as successful (*y = 1*) if the individual survived and failed (*y = 0*) otherwise. We specified a model using data from GPS and VHF collars across the overall reproductive season, restricting encounter histories to mid-March through end of August (days of year 75–242). This model definition followed Eq. 6, using a daily unit interval. We randomly sampled one location per individual per day from GPS data to align with the specified model structure. We assessed evidence for candidate environmental covariates (Appendix S5), plus covariates for sex (male/female), age, translocation status, transmitter type, and day of season. We also tested interactions between day of year and precipitation/drought covariates and used Bayesian variable selection (Appendix S5) to identify appropriate representations for the most influential covariates.

### Model implementation and validation

We fit hierarchical models using MCMC algorithms in NIMBLE^77,78^ with R 4.1.3^68^. We used high performance computing resources^79^ to fit lengthy model runs with large datasets. We specified vague or shrinkage priors for all estimated model parameters. Prior distributions used in modeling are reported in Appendix S6 and code is available in supplemental code^80^. We used 50,000 MCMC iterations, a 25,000 iteration burn-in, and retained every 10th posterior draw, provided convergence was acceptable. We examined chains visually and calculated Gelman-Rubin statistics^81^ to verify chain convergence ($\:\widehat{r\:}$< 1.1). For the variable selection population SSM, we ultimately used 100,000 iterations with a 50,000 burn-in and retained every 20th posterior draw. We calculated Bayesian *p*-values from posterior predictive distributions to evaluate goodness of fit, where values approaching 0 or 1 imply lack-of-fit^82^. Covariate effects were only described in the results section if they were carried forward from the variable selection stage. Then, we reported mean values of posterior distribution and 95% credible intervals (CRI) and strength of evidence for selected covariate effects using CRI overlap of zero and probability of direction (*pd*; proportion of posterior distribution with same sign as its median)^83^. We provide code^80^ and data^84^ to fit our models through USGS GitLab and ScienceBase.

## Results

### Population state-space model

**Fig. 1:**
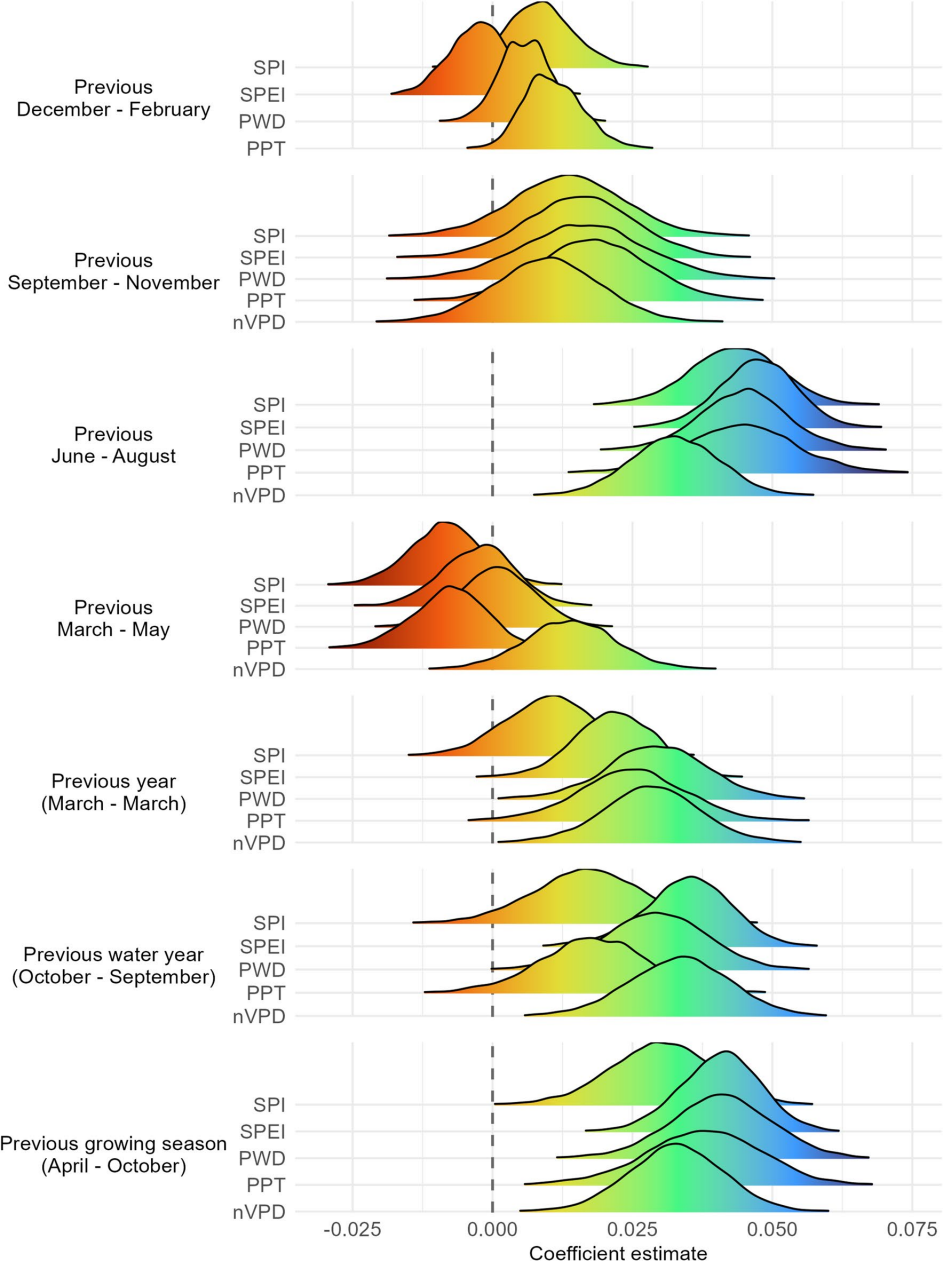
Climate coefficient estimates from 34 models of greater sage-grouse inter-annual population growth rates in response to environmental covariates in the Great Basin, USA, 1986–2021, including metrics representing average climate conditions across multiple time periods representing 0–1.5 years prior to the lek count for a current year. Climate metrics included Standardized Precipitation Index (SPI), Standardized Precipitation Evapotranspiration Index (SPEI), Potential Water Deficit (PWD), Precipitation (PPT), and negative Vapor Pressure Deficit (nVPD). Positive coefficient values (green to blue) indicate higher growth rates were associated with greater precipitation or moisture, and vice versa (orange to red indicate lower growth rates associated with greater precipitation or moisture).

 After filtering, we included 1,191 leks in our population SSM. Of 27 environmental covariates considered in the first modeling stage, we identified 7 potentially influential baseline covariates: elevation (radius [*r*] = 10 km), percent sagebrush cover (*r* = 5 km), percent annual forb and grass cover (*r* = 10 km), percent non-sagebrush shrub cover (*r* = 10 km), perennial forb and grass cover (*r =* 5 km), tree cover (*r =* 2.5 km), and impervious developed area (*r =* 2.5 km). Percent shrub cover and percent bare ground were confounded with other covariates, so we omitted these from final models. From model runs containing baseline covariates along with weather covariates, we observed that weather covariate influences on sage-grouse population growth rates depended on metric and time lag. The most explanatory model included a positive effect of PPT from the previous growing season (PPT_ngs_; Appendix S7-Table S1), wherein each additional cm of growing season precipitation predicted an increase in $\:\widehat{\lambda\:}$ of 0.61%, on average. Models that included SPEI during the previous water year and PWD during the previous summer were also highly ranked and had positive effects (Appendix S7-Table S1). Estimated precipitation effects on sage-grouse population growth were largely positive when averaged across multiple time lags, such that when more precipitation occurred during the previous summer, growing season, entire year, and/or water year, apparent abundance increased the following spring (Fig. 1). Moisture effects during fall and winter were less apparent (Fig. 1).

**Fig. 2:**
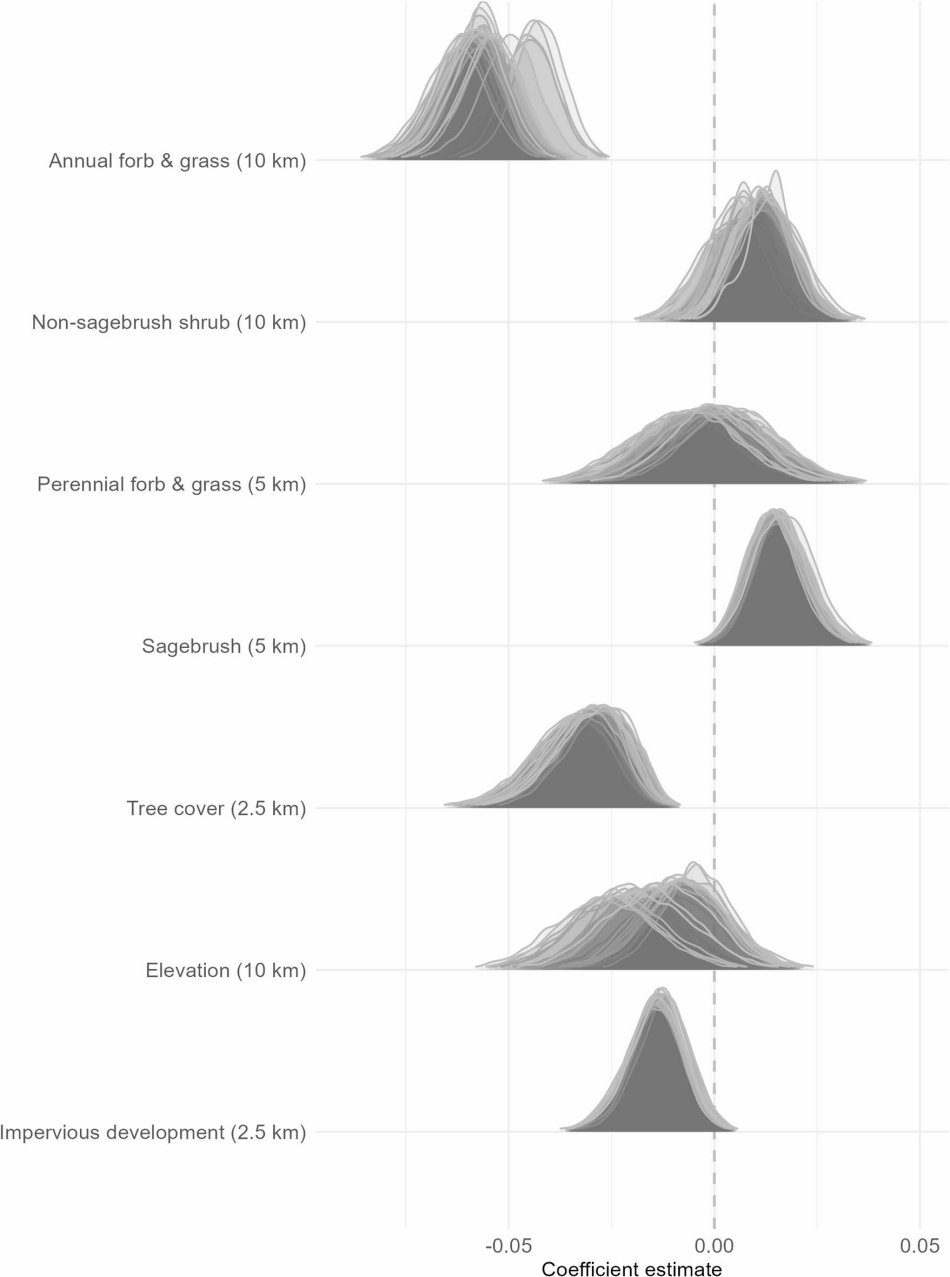
Coefficient estimates from 34 models of greater sage-grouse population growth rates in response to environmental covariates in the Great Basin, USA, 1986–2021. Each model included 7 environmental covariates predicted to influence sage-grouse populations, while testing the effect of 1 of 34 climate metrics. Each curve represents the posterior distribution of the coefficient estimate from a single model run for each of the 34 climate covariates. Positive coefficient values indicate higher growth rates with higher values of the covariate, and vice versa.

Accounting for weather covariates, sage-grouse population growth rates were positively associated with sagebrush cover and negatively associated with annual forb and grass and tree cover (Fig. 2). More impervious developed area was also negatively associated with growth rates, while we found less evidence for other covariates (Fig. 2).

### Nest survival

Of 1,794 sage-grouse nests monitored across 24 field sites, 2003–2021, 889 failed and 905 hatched eggs. Nesting females were primarily adults (≥ 2 years = 71%). Bayesian variable selection methods identified seven influential covariates to include in the final nest survival model describing day of season, percent cover of sagebrush (*r =* 75 m), non-sagebrush shrub (*r* = 1,451 m), annual forb and grass (*r =* 75 m), bare ground (*r =* 167 m), topographic roughness (*r* = 439 m), and VPD. We did not detect evidence for interactions. Evidence for selected covariates is reported in Appendix S8- Table S1.

**Fig. 3:**
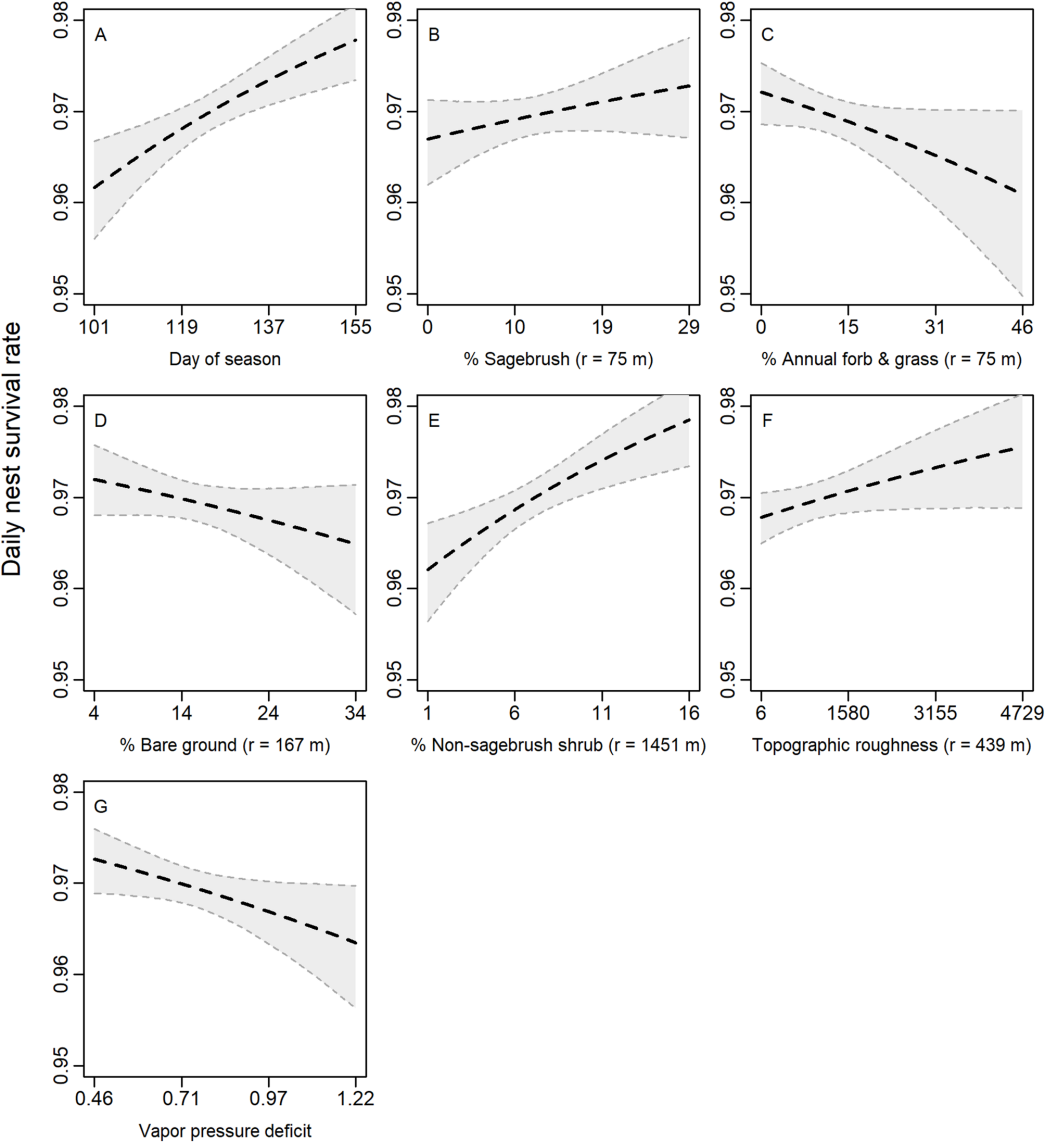
Predicted daily survival rate of greater sage-grouse nests in response to environmental covariates measured in the Great Basin region of the USA, 2003–2021. Effects were estimated using a hierarchical logistic exposure model in a Bayesian framework with variable and scale selection techniques, where the radius (r) of the most informative scale of measurement is indicated in meters. Heavy dashed lines (black), represent the median of the posterior distribution for each parameter estimate, while gray shadings represent the 95% credible interval.

Cumulative (38-day) nest survival probability was 0.308 (95% CRI = 0.284–0.333). Our final model converged, exhibited good fit (Bayesian *P* = 0.623, all $\:\widehat{r\:}$<1.1), and demonstrated strong evidence (i.e., 95% CRI not overlapping 0) that sage-grouse nests exhibited higher survival with increasing day of season and more non-sagebrush shrub cover and decreased survival with higher VPD (Table 1; Fig. 3). We observed moderate evidence (95% CRI overlapped 0, but *pd* > 0.85) that nest survival was higher with greater topographic roughness and more sagebrush cover but lower with increasing bare ground and annual forb and grass cover (Fig. 3). Results are summarized in Table 1.

**Table 1:** Posterior distributions of environmental covariate effects and derived estimates of greater sage-grouse nest survival estimated in the Great Basin region of the USA, 2003–2021. Parameters were estimated using a hierarchical logistic exposure model in a Bayesian framework with variable and scale selection techniques. Posterior distributions are characterized by mean, standard deviation (SD), 2.5th percentile, 50th percentile, and 97.5th percentile. *R-hat* values < 1.1 indicate parameter convergence, and *Pd* describes the proportion of the posterior distribution occurring on the same side of 0 as the mean.

Parameter	Description	Mean	SD	2.5th	50th	97.5th	*R*-hat	Pd
*β*_*0*_	Intercept	3.460	0.035	3.390	3.460	3.530	1.000	1.000
*β*_*1*_	Day of season	0.146	0.040	0.069	0.146	0.224	1.000	1.000
*β*_*2*_	Sagebrush cover[1]	0.054	0.045	−0.029	0.053	0.144	1.000	0.890
*β*_*3*_	Annual forb & grass cover[1]	−0.089	0.049	−0.184	−0.089	0.005	1.000	0.970
*β*_*4*_	Bare ground cover[2]	−0.063	0.045	−0.151	−0.063	0.021	1.000	0.920
*β*_*5*_	Non-sagebrush shrub cover[6]	0.159	0.047	0.067	0.158	0.250	1.000	1.000
*β*_*6*_	Topographic roughness[5]	0.076	0.042	−0.003	0.075	0.159	1.000	0.970
*β*_*7*_	Vapor pressure deficit	−0.079	0.040	−0.157	−0.079	−0.001	1.000	0.980
Λ	Shrinkage rate parameter	7.760	1.590	4.200	8.020	9.910	1.000	NA
*Derived estimates*								
*DSR*	Daily nest survival	0.969	0.001	0.967	0.969	0.971	NA	NA
*S*	38-day nest survival	0.308	0.013	0.284	0.308	0.333	NA	NA
*P*	Bayesian P-value	0.623						

Numbers in brackets represent the model-selected scale of analysis (i.e., circular moving window radius *r*) ordered by size, where 1–6 denote *r* = 75, 167, 260, 370, 439, and 1,439 m, respectively. Letters in brackets similarly represent the α value for an exponential distance decay function, where a–f denote α = 75, 167, 260, 370, 439, and 1,439 m, respectively.

### Brood survival

 Of 828 sage-grouse broods tracked from hatch up to 50 days across 23 field sites, 2003–2021, 353 failed prior to 50 days and 475 succeeded. Brood-rearing females were primarily adults (age ≥ 2 years at nest initiation = 71.8%). Bayesian variable selection methods identified 14 influential covariates to include in the final brood survival model describing day of season, age of brood, hen age, percent sagebrush cover (*r =* 370 m), percent non-sagebrush shrub cover (*r* = 1,451 m), percent bare ground (*r* = 1,451 m), percent tree cover (*r* = 1,451 m), topographic roughness (*r* = 167 m), transformed aspect (*r* = 1,451 m), proximity to seasonal wetland (*α =* 260 m), mean minimum temperature (TMIN), standardized precipitation during March–May of the current year (SPI_mm_), and standardized precipitation during the previous September–November (SPI_sn_). Evidence for selected covariates and scales are reported in Appendix S8-Table S2.

**Fig. 4:**
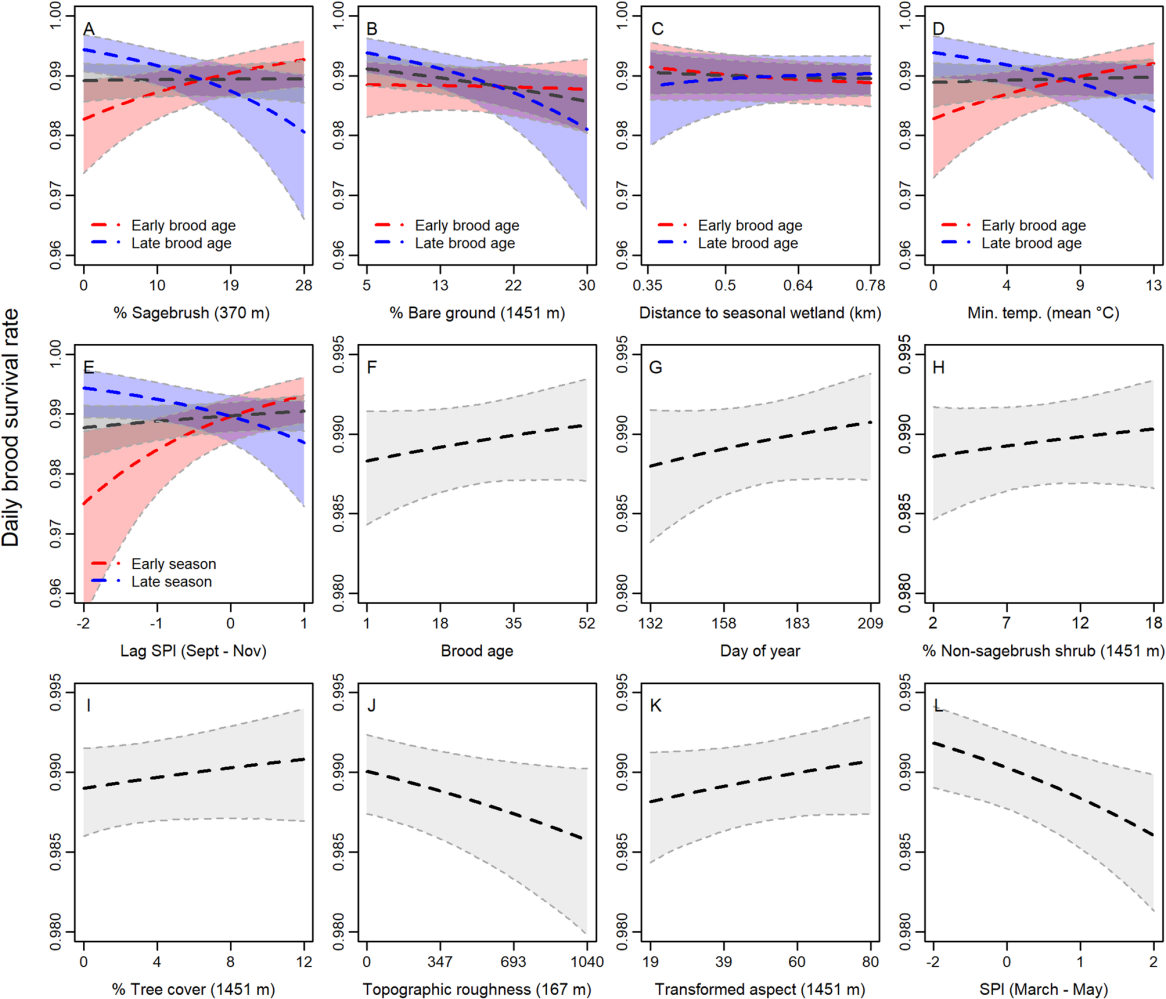
Predicted daily survival rate of greater sage-grouse broods in response to environmental covariates measured in the Great Basin region of the USA, 2003–2021. Effects were estimated using a hierarchical logistic exposure model in a Bayesian framework with variable and scale selection techniques, where the radius of the most informative scale of measurement is indicated in meters. Heavy dashed lines (black), represent the median of the posterior distribution for each parameter estimate, while gray shadings represent the 95% credible interval. Interaction effects are demonstrated in panels A–E, where red symbology indicates the effect for either early brood age or day of season (2.5th percentile) and blue symbology indicates the effect for late brood age or day of season (97.5th percentile).

Cumulative (50-day) brood survival probability was 0.564 (0.499–0.623). Our final model converged and exhibited good fit (Bayesian *P* = 0.612, all $\:\widehat{r\:}$<1.1). We observed strong evidence that sage-grouse broods had increased survival in areas with more sagebrush cover during early brood-rearing but reduced survival with more sagebrush cover and bare ground during the later brood-rearing stage (e.g., negative interaction between sagebrush cover and brood age, bare ground and brood age; Table 2; Fig. 4). Sage-grouse also exhibited negative survival responses to higher topographic roughness, and when SPI_mm_ was higher (Table 2; Fig. 4). We observed strong evidence for higher early brood survival when SPI_sn_ was higher and with higher minimum temperatures (TMIN), but the effects of SPI_sn_ and TMIN declined seasonally (negative interaction of SPI_sn_ with day of season and TMIN with brood age; Table 2; Fig. 4), with strong evidence that higher SPI_sn_ and TMIN reduced late brood-rearing survival. We observed moderate evidence that brood survival was higher for adult hens (vs. yearlings), with more tree cover, on predominantly southwest-facing slopes, and closer to seasonal wetlands during the early brood-rearing stage (negative interaction between proximity to wetland and brood age; Table 2; Fig. 4). We also observed weak evidence for higher survival as broods aged. Results are summarized in Table 2.

**Table 2:** Posterior distributions of environmental covariate effects and derived estimates of greater sage-grouse brood survival estimated in the Great Basin region of the USA, 2003–2021. Parameters were estimated using a hierarchical logistic exposure model in a Bayesian framework with variable and scale selection techniques. Posterior distributions are characterized by mean, standard deviation (SD), 2.5th percentile, 50th percentile, and 97.5th percentile. *R-hat* values < 1.1 indicate parameter convergence, and *Pd* describes the proportion of the posterior distribution occurring on the same side of 0 as the mean.

Parameter	Description	Mean	SD	2.5th	50th	97.5th	*R*-hat	Pd
*β*_*0*_	Intercept	4.470	0.101	4.270	4.470	4.660	1.000	1.000
*β*_*1*_	Sagebrush cover[4]	−0.011	0.053	−0.121	−0.010	0.093	1.000	0.580
*β*_*2*_	Non-sagebrush shrub cover[6]	0.047	0.062	−0.069	0.043	0.176	1.000	0.770
*β*_*3*_	Bare ground cover[6]	−0.136	0.051	−0.236	−0.136	−0.034	1.000	1.000
*β*_*4*_	Tree cover[6]	0.056	0.056	−0.045	0.051	0.176	1.000	0.840
*β*_*5*_	Topographic roughness[2]	−0.096	0.048	−0.188	−0.096	−0.002	1.000	0.980
*β*_*6*_	Transformed aspect[6]	0.061	0.051	−0.032	0.059	0.165	1.000	0.890
*β*_*7*_	Proximity to seasonal wetland[b]	0.055	0.071	−0.061	0.045	0.218	1.000	0.780
*β*_*8*_	Mean minimum temperature	0.007	0.060	−0.114	0.006	0.129	1.000	0.550
*β*_*9*_	SPI (Mar–May)	−0.165	0.062	−0.287	−0.165	−0.043	1.000	1.000
*β*_*10*_	SPI (Sept–Nov)	0.054	0.057	−0.051	0.051	0.172	1.000	0.830
*β*_*11*_	Sagebrush cover[4] × Brood age	−0.163	0.060	−0.279	−0.164	−0.044	1.000	1.000
*β*_*12*_	Bare ground cover[6] × Brood age	−0.083	0.051	−0.185	−0.082	0.010	1.000	0.960
*β*_*13*_	Proximity to seasonal wetland[b] × Brood age	−0.073	0.062	−0.202	−0.070	0.036	1.000	0.890
*β*_*14*_	Mean minimum temperature × Brood age	−0.131	0.059	−0.247	−0.131	−0.016	1.000	0.990
*β*_*15*_	SPI (Sept–Nov) × Day of season	−0.143	0.058	−0.257	−0.143	−0.031	1.000	1.000
*β*_*16*_	Day of season	0.070	0.070	−0.055	0.065	0.220	1.000	0.850
*β*_*17*_	Hen age	0.114	0.104	−0.062	0.105	0.337	1.000	0.870
*β*_*18*_	Brood age	0.064	0.065	−0.053	0.061	0.200	1.000	0.840
Λ	Shrinkage rate parameter	8.530	1.110	5.930	8.760	9.940	1.000	NA
*Derived estimates*								
*DSR*	Daily brood survival	0.989	0.001	0.986	0.989	0.991	NA	NA
*S*	50-day brood survival	0.564	0.032	0.499	0.565	0.623	NA	NA
*P*	Bayesian P-value	0.612						

Numbers in brackets represent the model-selected scale of analysis (i.e., circular moving window radius r) ordered by size, where 1–6 denote r = 75, 167, 260, 370, 439, and 1,439 m, respectively. Letters in brackets similarly represent the α value for an exponential distance decay function, where a–f denote α = 75, 167, 260, 370, 439, and 1,439 m, respectively.

### Adult and yearling survival

 We tracked 2,500 sage-grouse adults and yearlings during the reproductive season, across 26 field sites, 2003–2021. We recorded 625 mortalities. Our data comprised primarily adult females (age ≥ 2 years = 71.8%, female = 73.1%), including both nesting and non-nesting individuals. Bayesian variable selection identified 12 influential covariates to include in the final survival model describing day of season, sex, non-sagebrush shrub percent cover (*r* = 1,451 m), perennial forb and grass percent cover (*r* = 167 m), percent bare ground (*r* = 370 m), percent tree cover (*r* = 167 m), topographic position (*r* = 260 m), proximity to mesic rangeland (*α =* 439 m), proximity to wet meadow (*α =* 1,451 m), average VPD, average spring snow water equivalent (SWE), and previous growing season SPEI (SPEI_ngs_). Evidence for selected covariates and scales is reported in Appendix S8-Table S3.

**Fig. 5:**
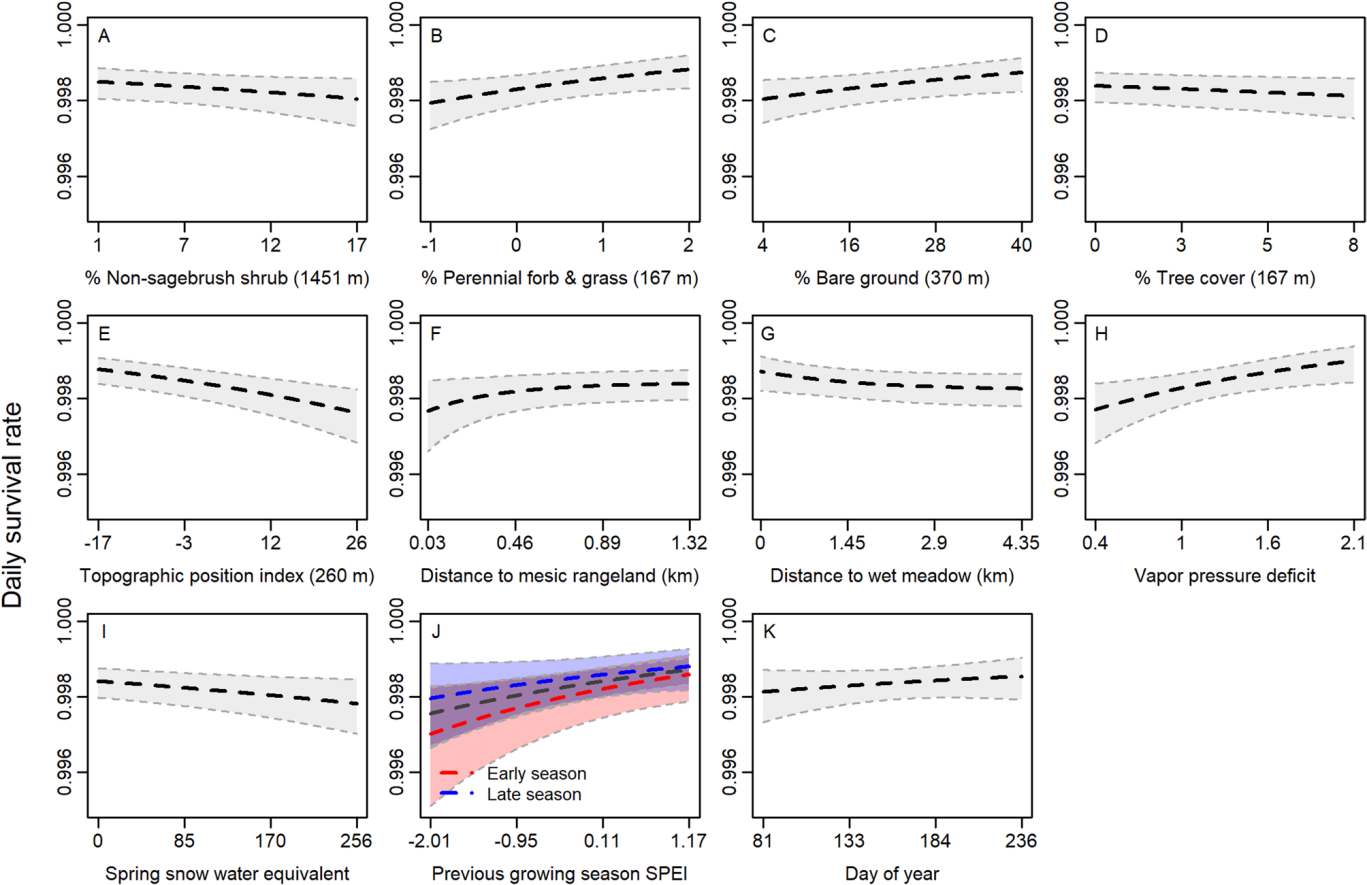
Predicted daily survival rate of greater sage-grouse adults and yearlings during their full reproductive period in response to environmental covariates measured in the Great Basin region of the USA, 2003–2021. Effects were estimated using a hierarchical logistic exposure model in a Bayesian framework with variable and scale selection techniques, where the radius of the most informative scale of measurement is indicated in meters. Heavy dashed lines (black), represent the median of the posterior distribution for each parameter estimate, while gray shadings represent the 95% credible interval. Interaction effects are demonstrated if supported by models, where red symbology indicates the effect for early brood day of season (2.5th percentile) and blue symbology indicates the effect for late day of season (97.5th percentile).

Cumulative (167-day) survival probability was 0.746 (0.698–0.784) spanning the reproductive season. Our final model converged and exhibited reasonable fit (Bayesian *P* = 0.775, all $\:\widehat{r\:}$<1.1). We observed strong evidence that sage-grouse exhibited increased survival with more perennial forb and grass cover, more bare ground, closer to wet meadow habitats, with higher average VPD, and with higher SPEI_ngs_. There was strong evidence of reduced survival with higher topographic position index, closer to mesic rangelands, and with higher spring SWE (Table 3; Fig. 5). Our model exhibited moderate evidence that survival decreased with more non-sagebrush shrub and tree cover (Table 3; Fig. 5). Evidence in the final model was more uncertain for sex, day of season, and the interaction between SPEI_ngs_ and day of season. Results are summarized in Table 3; Fig. 5.

**Table 3:** Posterior distributions of environmental covariate effects and derived estimates of greater sage-grouse adult and yearling survival estimated in the Great Basin region of the USA, 2003–2021. Parameters were estimated using a hierarchical logistic exposure model in a Bayesian framework with variable and scale selection techniques. Posterior distributions are characterized by mean, standard deviation (SD), 2.5th percentile, 50th percentile, and 97.5th percentile. *R-hat* values < 1.1 indicate parameter convergence, and *Pd* describes the proportion of the posterior distribution occurring on the same side of 0 as the mean.

Parameter	Description	Mean	SD	2.5th	50th	97.5th	*R*-hat	Pd
*β*_*0*_	Intercept	6.350	0.100	6.140	6.350	6.530	1.010	1.000
*β*_*1*_	Non-sagebrush shrub cover[6]	−0.069	0.045	−0.157	−0.069	0.016	1.000	0.940
*β*_*2*_	Perennial forb & grass cover[2]	0.166	0.070	0.032	0.166	0.306	1.000	0.990
*β*_*3*_	Bare ground cover[4]	0.126	0.061	0.008	0.127	0.247	1.000	0.980
*β*_*4*_	Tree cover[2]	−0.048	0.029	−0.102	−0.049	0.009	1.000	0.950
*β*_*5*_	Topographic position index[3]	−0.149	0.033	−0.213	−0.149	−0.083	1.000	1.000
*β*_*6*_	Proximity to mesic rangeland[e]	−0.100	0.048	−0.192	−0.100	−0.005	1.000	0.980
*β*_*7*_	Proximity to wet meadow[f]	0.101	0.052	0.003	0.100	0.205	1.000	0.980
*β*_*8*_	Vapor pressure deficit	0.252	0.097	0.053	0.255	0.438	1.000	1.000
*β*_*9*_	Snow water equivalent	−0.091	0.037	−0.161	−0.091	−0.017	1.000	0.990
*β*_*10*_	SPEI (previous growing season)	0.164	0.044	0.076	0.164	0.250	1.000	1.000
*β*_*11*_	SPEI (previous growing season) × Day of season	−0.018	0.039	−0.096	−0.016	0.056	1.000	0.670
*β*_*12*_	Day of season	0.071	0.081	−0.077	0.064	0.245	1.000	0.820
*β*_*13*_	Sex	0.090	0.103	−0.089	0.080	0.310	1.000	0.810
Λ	Shrinkage rate parameter	7.800	1.460	4.650	8.000	9.890	1.000	1.000
*Derived estimates*								
*DSR*	Daily adult survival	0.9982	0.0002	0.9978	0.9983	0.9985	NA	NA
*S*	Seasonal adult survival	0.746	0.022	0.698	0.748	0.784	NA	NA
*P*	Bayesian P-value	0.775						

Numbers in brackets represent the model-selected scale of analysis (i.e., circular moving window radius r) ordered by size, where 1–6 denote r = 75, 167, 260, 370, 439, and 1,439 m, respectively. Letters in brackets similarly represent the α value for an exponential distance decay function, where a–f denote α = 75, 167, 260, 370, 439, and 1,439 m, respectively.

## Discussion

**Fig. 6:**
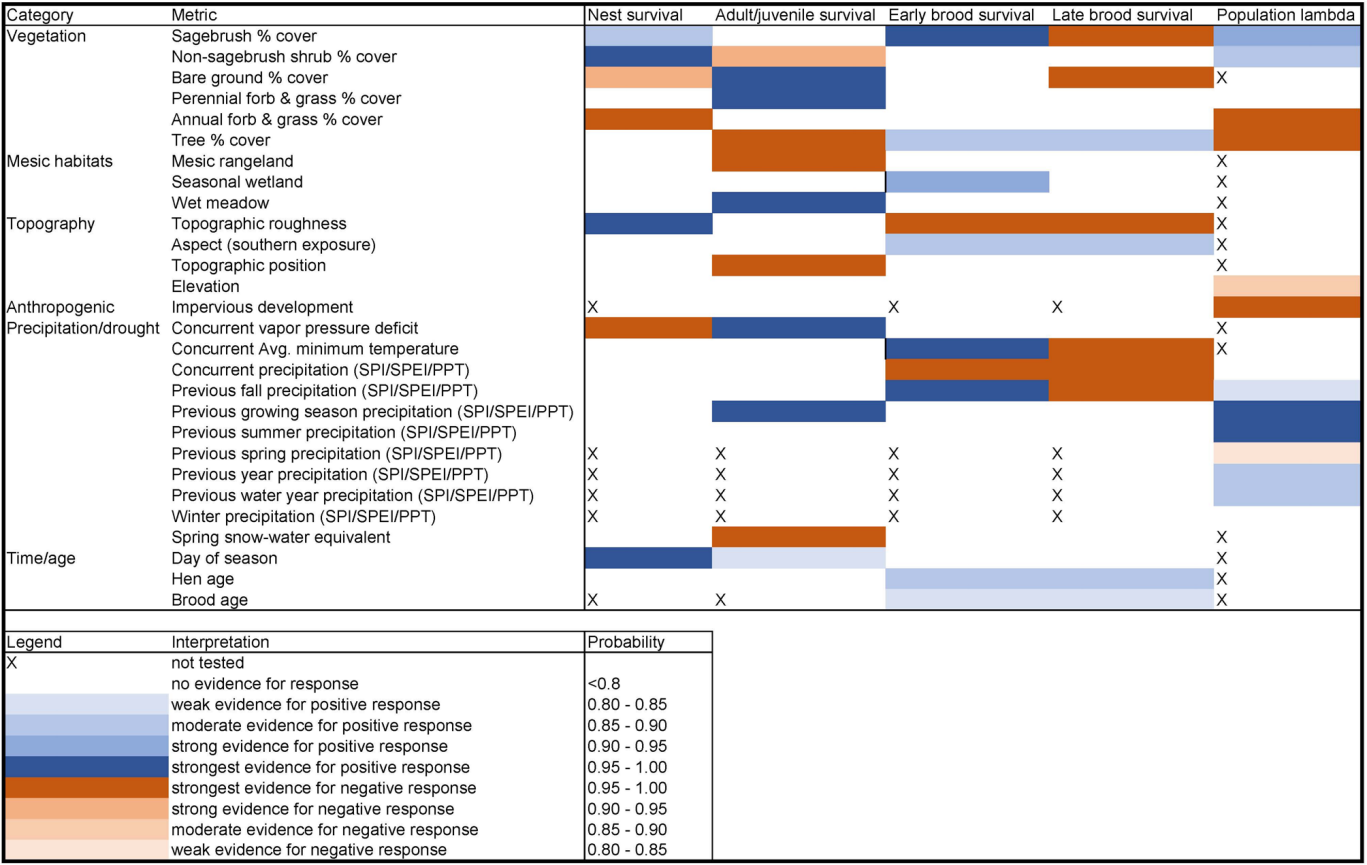
Visual summary of individual and environmental influences (e.g., landcover, vegetation, topography, precipitation and/or moisture) supported by hierarchical models of greater sage-grouse nest survival, brood survival, adult survival, and population rate of change (*λ*) in the Great Basin, USA, 1986–2021. Precipitation effects are represented by Standardized Precipitation Index (SPI), Standardized Precipitation Evapotranspiration Index (SPEI), or cumulative precipitation (PPT). The generalized effect for each variable is represented by color coding, where a gradient of blue shades indicates the range of support for positive effects from weak (lighter shade) to strong (darker shade), whereas orange shades indicate range of support for negative effects from weak (lighter shade) to strong (darker shade).

Our retrospective analyses leveraged multiple data types collected over 35 years to identify biotic and abiotic factors, including weather and climate, that influence imperiled sage-grouse population dynamics across the Great Basin, a region that is susceptible to a hotter and potentially drier future^85^. At the population level, our results corroborate previously documented patterns relating precipitation, drought, and aridity to population performance^18–20,45^. By considering multiple time lags and separately examining the responses of vital rates (i.e., nest survival, brood survival, adult survival), our study provides additional context for identifying the mechanistic processes through which such effects operate. Our results indicate that moisture availability and precipitation patterns can impose both positive and negative influences on different vital rates depending on the timing and life-stages affected. However, the net effects of moisture on apparent population growth rate (and its corresponding rate of change, *λ*), were positive. By considering landcover influences alongside measures of precipitation and moisture, we further documented a comprehensive suite of effects on sage-grouse demographic rates and *λ* (Fig. 6, Appendix S9) that may be affected by future climate conditions and changing disturbance regimes^32,86,87^. Precipitation and moisture were directly associated with *λ*, based on indices spanning time lags up to 1.5 years prior to annual lek counts. Lag effects likely result in part from the influence of resource availability during reproductive life stages, including forb and associated arthropod communities^22,88^, ultimately leading to population recruitment that is realized the following year. Lags may further reflect cumulative influences of soil moisture on herbaceous growth and resulting protective cover^42,43^, with longer lags suggesting a key role of residual grass cover in providing concealment for nests^42,89^.

We found additional support for hypothesized habitat-mediated effects of cumulative moisture conditions on all three vital rates. The strongest positive weather effects on *λ* were associated with precipitation falling during the previous growing season, including summer when the Great Basin typically experiences a seasonal drought that may constrain chick growth and survival^60,90–92^. Summer and growing season precipitation may delay the desiccation of succulent forbs and other herbaceous vegetation^91^ and provide sustained diet resources that promote brood habitat selection and survival^93^. Further, cumulative effects of growing season precipitation likely minimize risky seasonal movements in search of suitable brood-rearing habitat and important forage^94,95^. Although we did not identify an effect of previous summer precipitation on brood survival^91,85,92,93^, we did find a strong positive association between brood survival and previous fall precipitation, and the relative magnitude of this effect was the largest we detected on any vital rate (see Appendix S10 and Fig. 6). Fall precipitation may help sustain soil moisture and contribute to early-season snowpack that recharges mesic resources and supports primary productivity during the subsequent growing and brood-rearing seasons^39,60^. Why the effect of previous-fall precipitation on brood survival transitioned from positive during the early brood period to negative during the late brood period is unclear but could reflect mechanisms related to density dependent brood survival or mismatches between early and late brood-rearing habitat requirements. We also acknowledge inherent difficulties in measuring brood and/or chick survival^96^, which can limit the ability of the data to fully represent underlying dynamics. While individual chick survival might better reflect overall recruitment contributions to population growth^90,97^, such data are difficult to acquire without marking chicks, which can have detrimental effects on their survival^98^.

We also observed negative effects of drought indices on nest and adult survival, at different temporal scales. Nest survival declined with increasing concurrent monthly VPD. Although short-term vegetation responses to moisture may be somewhat limited, more-instantaneous responses by arthropods or forbs may enhance nesting female nutrition and foraging efficiency^46,47^. Furthermore, moisture availability during the nest period may simply reflect phases in general wet-dry cycles^99^ that affect longer-term primary productivity and vegetation dynamics that mediate nest concealment^42^. Adult survival also increased with more previous growing season moisture (SPEI), which may be associated with greater residual grass cover, enhanced predator avoidance, and carryover effects on adult body condition^19,44,100^. Collectively, these influences of moisture and aridity on each of three vital rates help to corroborate the population-level responses apparent in the SSM and inform our understanding of the underlying mechanisms.

Interestingly, we observed that adult survival increased with greater concurrent spring-summer aridity. However, this finding may be reconciled when considered through the lens of life-history optimization^101^. Sage-grouse exhibit a relatively slow life-history strategy that prioritizes adult survival, longevity, and subsequent lifetime reproductive success above individual reproductive attempts^102^, the success of which are likely more sensitive to interannual climate variability^19,22,24^. Therefore, sage-grouse more often forego nesting^103–105^, exhibit lower renesting propensity^106^, and lay smaller clutches^107^ during drought periods. Collectively, these effects may reduce the costs of reproduction, and successful reproduction has been associated with reduced sage-grouse survival during the following summer and fall^91^. Similarly, the negative effect of spring snowpack we detected on adult survival could reflect increased reproductive effort and costs in favorable years or could be associated with delayed plant phenology and reduced access to forage resources^108–110^.

In addition to lagged and cumulative effects mediated by vegetation responses to soil-moisture, sage-grouse may experience acute physiological stress from exposure to inclement weather, especially among more sensitive life-stages^27,39,111^. We found support for the hypothesis that concurrent spring precipitation imposes acute negative effects on brood survival, corroborating previous studies showing reduced sage-grouse productivity coinciding with heavy spring precipitation^110,112^. In addition to possible hypothermia effects^27^, precipitation around the time of hatching could lead to olfactory detection of nests and broods by predators^113,114^, reduced foraging opportunities among pre-thermoregulatory chicks^28,115^, and delayed plant phenology, especially when accompanied by cold temperatures^108^. We also observed lower survival of young broods during periods of lower minimum temperatures. That effect was reversed among older broods, that are better able to thermoregulate^111^ and for which lower minimum temperature might instead be associated with lower heat stress or delayed desiccation of succulent vegetation during summer^39,94^. We did not detect hypothesized negative exposure effects of precipitation or cold conditions on nest survival, unlike previous studies conducted in wetter ecoregions or that considered multi-day precipitation events^26,114^.

Sage-grouse *λ* is thought to be sensitive to adult female survival, nest survival^23,24,116^, and in some areas, chick survival^23,24^. Our population model results suggest that a combination of vital rates was positively associated with prior growing season moisture conditions, implying that survival and recruitment largely benefit from moisture availability. Our models of nest, adult, and brood survival were mixed in their support for the same idea, as positive vs. negative precipitation and/or moisture availability effects were not always consistent with the larger population model results. For example, while our nest survival results supported positive precipitation contributions to *λ*, our brood and adult survival results only partially supported this, with some effects being seemingly contradictory. Nonetheless, the effects of moisture availability with greatest magnitude (Appendix S10) appeared to be those that supported positive contributions to *λ*, apparently outweighing the observed negative effects. Such nuanced results can often be reconciled through careful consideration of individual mechanisms by which different moisture regimes affect demographic processes. We further emphasize that our analyses of three independent vital rates cannot provide a complete picture of all contributions affecting *λ*, and that such specific vital rate contributions are almost certainly tied to density-dependent mechanisms and thereby likely to vary across space and time. As such, we consider our larger pattern-based model results to be a more reliable indicator of the broad scale precipitation effects on sage-grouse populations, while specific mechanisms would continue to benefit from additional study.

Lek counts are assumed to accurately reflect demographic processes related to birth and death rates, but that assumption may not always be met across shorter temporal scales because lek counts are sensitive to variation in male attendance^49,56^ and detection^117^. In addition to age-based variation in the rate and timing of lek attendance^118^, male lek attendance may be reduced or delayed in high-snow years, or mating may occur away from traditional lek sites if they are not accessible^56,119^. Our SSM may therefore underestimate *λ* in years of high snowpack or when the population’s age structure shifts towards a higher proportion of yearlings. This mechanism may partially explain why *λ* showed relatively low sensitivity to winter precipitation and moisture. Although winter snowpack is expected to have a positive effect on subsequent chick survival and recruitment^39,60^, these effects would not propagate to measured population growth until the following year^45^.

We identified multiple pathways mediating the documented associations between moisture availability, aridity, and sage-grouse population performance. Although projected future precipitation regimes are less certain than those of increasing temperature, rising temperatures would increase evaporative demand, particularly during the hottest part of the year, reduce snowpacks and subsequent recharge of mesic resources, and limit future soil moisture availability with the net effects of exacerbated ecological drought conditions in the Great Basin^30^. By developing models of how weather and climate influence the demography and population trends of sage-grouse, we lay the groundwork for projecting population performance from models of future climate conditions^120^. Spatially explicit projections of future population performance could guide future climate-informed adaptive management efforts following, for example, the resist-accept-direct (RAD) framework^121^, with extirpation probabilities helping to identify areas where sage-grouse may be threatened under future conditions. Alternatively, some populations may benefit from future climate conditions and represent priorities for habitat preservation and improvement. Within areas where habitats are expected to remain climatically suitable, our results can inform management strategies that mitigate future effects by better identifying the mechanisms through which drought and extreme weather affect sage-grouse populations. For example, the importance of lagged effects of moisture conditions occurring in previous seasons on population growth, adult, and brood survival may suggest managing for residual grass cover, which could be most effective in xeric regions^42^. Moreover, the multiple pathways by which aridity limits sage-grouse populations suggest this species may benefit from management strategies focused on restoring hydrologic function^122,123^. Similarly, it may be desirable to maximize native plant communities’ access to available moisture to offset expected increases in atmospheric demand, decreasing snowpacks, and intensifying ecological drought conditions. This may be particularly true in mesic areas where resources must persist through late summer and fall to support chick survival and recruitment^39,40,60^.

Our research reinforces the importance of large-scale conservation initiatives designed to prioritize and preserve the integrity of sagebrush ecosystems and the unique species they support^12,124,125^. Threats to obligate species within these systems often mirror a larger pattern of habitat degradation, wherein changing climate interacts with anthropogenically altered landscapes, often driving plant community feedback cycles that are predominantly harmful to native species^18,86,126^. For example, a warming climate, especially when characterized by more intense cycles of precipitation and drought, may favor continued spread of invasive annual grasses that impose ecological drought conditions^127,128^. Combating expansion of such species would likely reduce the negative effects of intensifying drought cycles on native wildlife. By furthering knowledge of sage-grouse sensitivity to weather and climate patterns, our study informs conservation efforts for a warmer and potentially drier future.

## Supplementary Information

Below is the link to the electronic supplementary material.

Supplementary Material 1

## Data Availability

Data and code supporting the results and conclusions of this study are archived at USGS ScienceBase (10.5066/P14QJU9C) and GitLab (10.5066/P177TRXQ).
