# Supplementary material for: Precipitation timing mediates life-stage and population-level associations with climate for an indicator species
Source: Sci Rep. 2025 Oct 23;15:37051. doi: 10.1038/s41598-025-20796-y (PMC12549991; doi:10.1038/s41598-025-20796-y)
Supplement: Supplementary file 1 — Supplementary Material 1 [file 41598_2025_20796_MOESM1_ESM.pdf]

Supplementary information supporting the manuscript:

O'Neil, S. T.[1*], C. G. Lundblad[2], B. E. Brussee[2], J. C. Tull[3], M. L. Casazza[1], J. R. Small[4], C. L. Aldridge[5], and P. S. Coates[1]. Precipitation timing mediates life-stage and population-level associations with climate for an indicator species. Scientific Reports.

[1] *U.S. Geological Survey, Western Ecological Research Center, Dixon, California, USA*

[2] *U.S. Geological Survey, Western Ecological Research Center, Reno, Nevada, USA*

[3] *U.S. Fish and Wildlife Science, Science Applications, Region 8, Reno, Nevada, USA*

[4] *Nevada Department of Wildlife, Reno, Nevada, USA*

[5] *U.S. Geological Survey, Fort Collins Science Center, Fort Collins, Colorado, USA*

**Corresponding: soneil@usgs.gov*

Any use of trade, firm, or product names is for descriptive purposes only and does not imply endorsement by the U.S. Government.

APPENDIX S1

Study area

The study area (Figure S1) comprised elevations -86–4,412 m, typically featuring hot dry summers, cold often-snowy winters, and variation along latitudinal, longitudinal, and altitudinal gradients. From observed lek locations, sage-grouse occupied elevations ~790–3,030 m (median = 1,766 m). Maximum summer (June–August) temperatures ranged from ~12–46° C and minimum temperatures ranged from ~8–40° C, demonstrating variation from hot summer desert conditions below sea level (Death Valley, CA) to cool high montane summits above 3,000 m. Most of annual precipitation typically falls during the winter – spring with more moisture prevailing at higher elevations, although summertime convective precipitation is more common across the southern portions. Droughts are periodic, and the precipitation regime is characterized by high interannual and interdecadal cycles between wet and dry periods[1]. The resulting vegetation, in areas typically occupied by sage-grouse, is characterized by the dominance of several species of sagebrush (*Artemisia* spp.) in the overstory, although other common species of shrubs include rabbitbrush (*Chrysothamnus* spp., *Ericameria* spp.), antelope bitterbrush (*Purshia tridentata*). Woodlands including juniper (*Juniperus* spp.) and single-leaf pinyon pine (*Pinus monophyla*) are present at higher elevations and have undergone modern expansion into sagebrush-dominated shrubland[2]. The herbaceous understory of sagebrush shrublands features native perennial grasses and forbs, but some areas are increasingly dominated by exotic annual grasses including cheatgrass (*Bromus tectorum*)[3–5]. These annual grasses create relatively continuous herbaceous cover that increases fire risk and decreases fire return intervals. Wildfires, in turn, remove sagebrush overstory and create landscapes prone to additional annual grass invasion[6] thereby perpetuating higher probability of future burns as part of a positive feedback cycle.

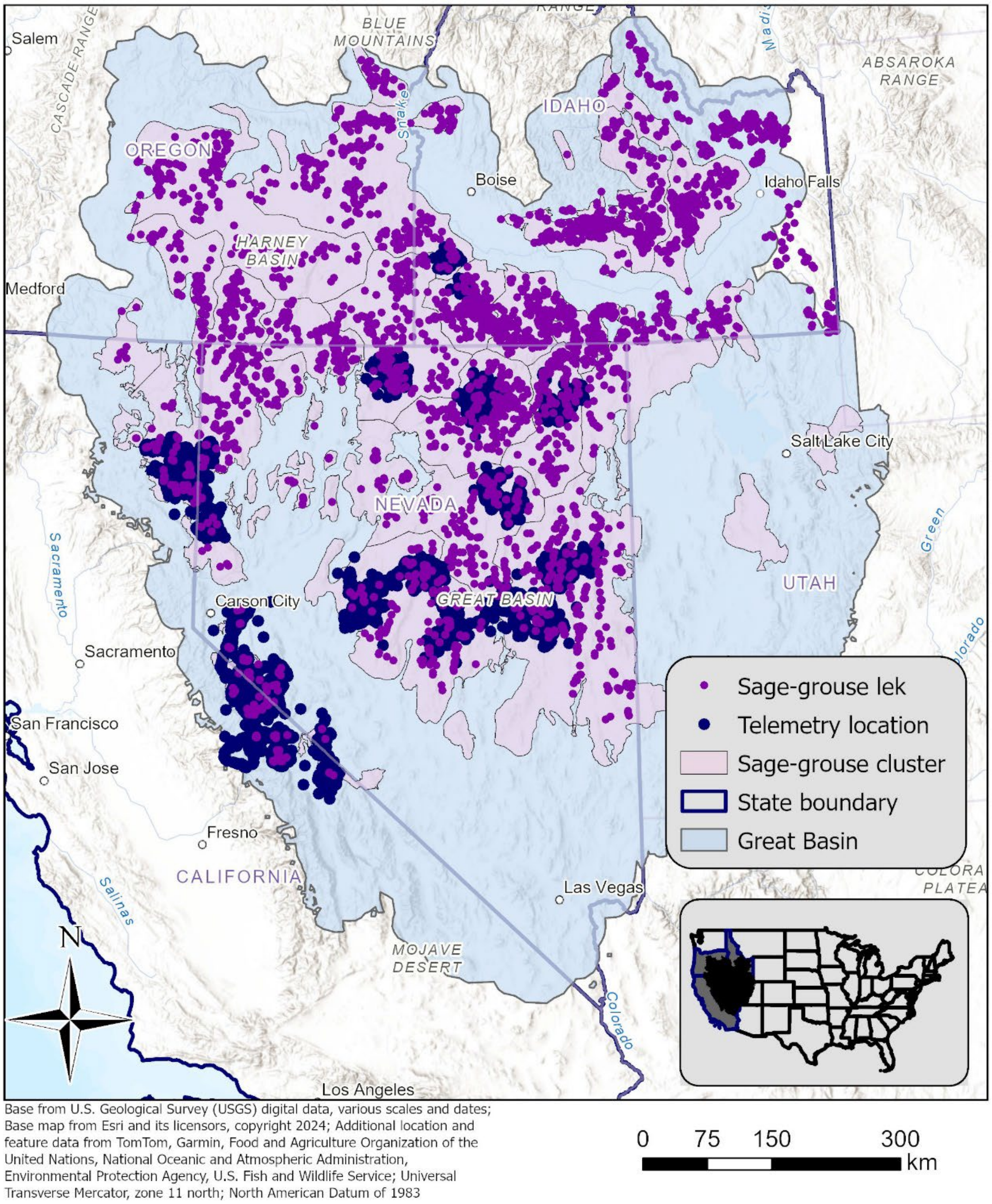

Figure S1. Locations of greater sage-grouse (*Centrocercus urophasianus*) from VHF radio and GPS telemetry (dark blue) and breeding lek sites (purple) used for studying effects of precipitation and drought on sage-grouse populations in the western United States. Telemetry data were collected from 2003–2021 in California, Idaho, and Nevada, and lek count data used for analyses occurred in California, Idaho, Nevada, and Oregon, and that spanned 1985–2021. Sage-grouse sub-population clusters represent level-5 polygon boundaries described in Coates et al. (2021) and O'Donnell et al. (2021). While leks also occur in Utah, data could not be made available by the state for this analysis.

Literature cited

1. Neilson, R. P., Lenihan, J. M., Bachelet, D. & Drapek, R. J. Climate change implications for sagebrush ecosystems. in *Transactions of the... North American Wildlife and Natural Resources Conference* (2005).

2. Filippelli, S. K. *et al.* Monitoring pinyon-juniper cover and aboveground biomass across the Great Basin. *Environmental Research Letters* **15**, 25004 (2020).

3. Bradley, B. A. *et al.* Cheatgrass (Bromus tectorum) distribution in the intermountain Western United States and its relationship to fire frequency, seasonality, and ignitions. *Biological invasions* **20**, 1493–1506 (2018).

4. Williamson, M. A. *et al.* Fire, livestock grazing, topography, and precipitation affect occurrence and prevalence of cheatgrass (Bromus tectorum) in the central Great Basin, USA. *Biological Invasions* **22**, 663–680 (2020).

5. Chambers, J. C. *et al.* Resilience to stress and disturbance, and resistance to Bromus tectorum L. invasion in cold desert shrublands of western North America. *Ecosystems* **17**, 360–375 (2014).

6. Balch, J. K., Bradley, B. A., D'Antonio, C. M. & Gómez-Dans, J. Introduced annual grass increases regional fire activity across the arid western USA (1980–2009). *Global Change Biology* **19**, 173–183 (2013).

APPENDIX S2

Lek filters used in hierarchical state space models of sage-grouse population growth

We used the Western Association of Fish and Wildlife Agencies (WAFWA) standardized sage-grouse lek database to model maximum annual counts of sage-grouse at leks. This database and its associated standardized protocols are described in Coates et al.[1] and O'Donnell et al.[2] We used the 2022 version of the database and subset it to include the years 1985–2021 for the states of California, Idaho, Nevada, and Oregon. This time period was chosen to align with available remotely sensed data and to approximately match a nadir-to-nadir cycling pattern described in Coates et al.[1] In addition to the protocols described in O'Donnell et al.[2], we applied the following filters:

1) Removed any lek counts that occurred outside of primary breeding period (1, Mar – 31, May).
2) Removed any lek counts that occurred more than 0.5 h before sunrise or more than 1.5 h after sunrise.
3) Removed any lek count if it was not listed as one of following survey method type: {'Ground survey,' 'Ground route survey,' Aerial fixed-wing survey,' or 'Aerial helicopter survey.'}
4) Retained only the maximum count from each lek, for each year.
5) Retained only active leks, defined as leks that had at least 2 or more consecutive years of 2 or more males counted during the time series.
6) Retained only active leks that had at least 9 verified counts (~25%) recorded during the full time series.

Literature cited

1. Coates, P. S. *et al.* Range-Wide Greater Sage-Grouse Hierarchical Monitoring Framework—Implications for Defining Population Boundaries, Trend Estimation, and a Targeted Annual Warning System. *U.S. Geological Survey Open-File Report 2020–1154, 243 p.,* https://doi.org/10.3133/ofr20201154 (2021).

2. O'Donnell, M. S. *et al.* Synthesizing and analyzing long-term monitoring data: A greater sage-grouse case study. *Ecol Inform* **63**, (2021).

Appendix S3

Data collection — Sage-grouse demographic data

We used spotlighting[1,2] to capture sage-grouse at 25 field sites (Appendix S1—Fig. S1) during spring and fall, 2003–2021. Most sage-grouse were fitted with very high frequency (VHF) necklace-style transmitters[3], while a subset of individuals was fitted with GPS Platform Transmitter Terminals (GeoTrak, Inc., Apex, North Carolina). Ages were determined in the fall as juvenile (hatch year), yearling (second year), and adult (>second year), and restricted to adult and yearling during spring. We located nests visually throughout the spring season (March–June) following consecutive telemetry observations of a female at the same location. We relocated the female twice weekly to record status and nest success, which was determined by ≥1 egg hatched (successful) or evidence of depredation or abandonment (failed). Following hatch, females and their broods were relocated during night or day every 10 days up to 50 days[4]. Broods were considered active if ≥1 chicks were observed, or females exhibited behavior suggesting brood-rearing. Brood failure was confirmed with multiple follow-up night and day checks. All birds were flushed at 50 days to confirm brood presence or absence. Further details can be found in Brussee et al.[4] Adults and juveniles with VHF transmitters were monitored primarily via ground telemetry during the nest and brood-rearing season; individuals with GPS transmitters were additionally tracked remotely year-round provided transmitters remained active. Locations of mortality signals during winter were marked and transmitters were retrieved in spring to confirm mortality; the mortality date was then estimated from the initiation of a GPS 'cluster' (repeated fixes occurring at the same location). Supplemental fate and location information for VHF-collared individuals were obtained monthly from aerial surveys during the non-breeding seasons. A small proportion of females were translocated within the Bi-State Distinct Population Segment during 2017–2021[5]; we included an indicator term for these individuals to account for possible translocation effects on survival.

Literature cited

1. Giesen, K. M., Schoenberg, T. J. & Braun, C. E. Methods for trapping sage grouse in Colorado. *Wildl Soc Bull* 224–231 (1982).

2. Wakkinen, W. L., Reese, K. P., Connelly, J. W. & Fischer, R. A. An improved spotlighting technique for capturing sage-grouse. *Wildl Soc Bull* **20**, (1992).

3. Kolada, E. J., Sedinger, J. S. & Casazza, M. L. Nest site selection by greater sage-grouse in Mono County, California. *Journal of Wildlife Management* **73**, 1333–1340 (2009).

4. Brussee, B. E. *et al.* Invasion of annual grasses following wildfire corresponds to maladaptive habitat selection by a sagebrush ecosystem indicator species. *Glob Ecol Conserv* **37**, e02147 (2022).

5. Meyerpeter, M. B. *et al.* Field methods for translocating female greater sage-grouse (Centrocercus urophasianus) with their broods. *Wildl Soc Bull* **45**, 529–537 (2021).

APPENDIX S4

Description of environmental covariates

Environmental covariate categories considered in analyses included vegetation, topography, mesic areas, anthropogenic development, and drought/precipitation. Vegetation components included annually-varying percent cover of shrub, sagebrush, non-sagebrush shrub, perennial forb and grass, annual forb and grass, and bare ground[1–3]. Topography components included elevation, topographic roughness, topographic position, heat load index, and transformed aspect. Mesic components included seasonal wetlands, mesic rangelands, wet meadows, and riparian areas[4]. For development, we used all percent developed imperviousness[5]. Characterizations of drought and precipitation[6,7] included monthly and seasonal measures of average precipitation (PPT), standardized precipitation index (SPI), standardized precipitation evapotranspiration index (SPEI), potential water deficit (PWD), vapor pressure deficit (VPD), average winter (1 Dec – 28 Feb) and spring (1 Mar – 31 May) snow water equivalent (SWE), and temperature (daily minimums and maximums; TMIN, TMAX, respectively). Seasonal measures for PPT, SPI, SPEI, PWD, and VPD included averages over spring, summer, fall, winter, the entire calendar year, growing season, and water year (detailed in main text; see Figure S1 for temporal alignment with breeding lek counts).

We assumed that responses to all environmental covariates would be scale dependent[8]. We specified two sets of candidate spatial scales, one specific to life stage analyses and another specific to population level analysis. For life stage analyses, we defined six candidate distances of 75, 167, 260, 370, 439, and 1,451 m[9,10], to determine the radial buffer distance for circular moving windows (zonal means) characterizing vegetation cover and topography covariates, and the α value for exponential distance decay functions, exp $(-d/\alpha)$, used to represent proximity to the different mesic resource types. Drought and precipitation indices were not evaluated at different spatial scales because the grid cell size of the source data exceeded the largest candidate distance for life stage analysis. For population analyses, we specified larger candidate distances of 2.5, 5, and 10 km, to capture the cumulative influence of demographic processes (recruitment and mortality) and a plausible range of spatial distributions centered around active lek sites [11,12]. We did not consider distance to mesic resources in population analyses, as such distances are more relevant at the individual level. Candidate buffer sizes for population analyses were applied to all other environmental covariates using moving window analysis.

General hypotheses

Although responses to environmental conditions were expected to vary by life stage, we conceived general hypotheses about how sage-grouse survival was likely to associate with covariates across our analyses, based on prevailing knowledge about the species and its landscape requirements:

- **Vegetation**: We hypothesized that sage-grouse survival would, in general, be positively associated with higher cover amounts for native herbaceous and shrubby vegetation components (e.g., perennial grass, sagebrush, shrub), while associating negatively with predominantly non-native (e.g., annual grass, i.e. largely comprising cheatgrass [*Bromus tectorum*]), and sagebrush-encroaching (tree cover, i.e. driven predominantly by pinyon-juniper woodlands; possibly non-sagebrush shrub) vegetation. These hypotheses were largely driven by an expected positive association with native, undisturbed habitat conditions that provide access to high quality forage and cover across life stages.
- **Topography**: We hypothesized that sage-grouse survival would, in general, be positively associated with higher relative elevation and/or topographic roughness, as these attributes are

often associated with increased landscape complexity that provides greater access and/or juxtaposition with mesic resources that are needed during key life stages such as brood rearing. In addition, such attributes could reduce exposure to predators. In contrast, we hypothesized negative relationships between sage-grouse survival and topographic position, as higher topographic position values correspond to higher elevations relative to surrounding areas, such as ridges or summits, which are often used more prevalently by avian predators. Finally, we hypothesized positive relationships between transformed aspect and/or heat load index for survival during nesting and/or early brood rearing, as higher values indicate southwest-facing slopes that likely have quicker green-up and access to resources early in the season. However, we anticipated a possible switch in this relationship corresponding to later life stages.

- **Mesic resources**: We hypothesized that sage-grouse survival would associate positively with areas closer to mesic resources, particularly during the brood-rearing life stage. However, we also anticipated possible exceptions (e.g., negative relationships) for mesic resource areas that likely hold higher densities of predators, such as riparian areas.
- **Anthropogenic**: We hypothesized negative relationships between sage-grouse survival and percent developed impervious, where relevant. However, sage-grouse generally selected areas with very low percent developed values, so it was not possible to test this hypothesis across all life stages.
- **Precipitation or drought**: We generally hypothesized positive relationships between sage-grouse survival and higher precipitation indices, and negative relationships between sage-grouse survival and indices of drought. However, we anticipated possible exceptions based on timing of precipitation and/or drought relative to timing of key sage-grouse life stage events (see main text).

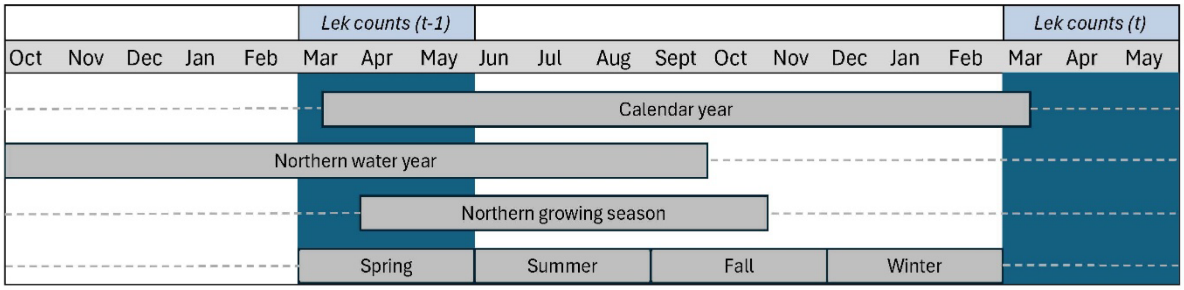

Figure S1. Graphical representation of alignment of seven different temporal lags considered for seasonal aggregation of precipitation or drought metrics, relative to Greater sage-grouse (*Centrocercus urophasianus*) male lek counts. Sage-grouse population change is measured based on the modeled change in apparent abundance, from the previous (year *t-1*) to current year (year *t*). Each temporal lag represents the cumulative or average conditions occurring over the corresponding time period, prior to the current year lek count, thus overlapping key important phases of sage-grouse life history that contribute to annual recruitment and survival.

Table S1. Candidate environmental covariates and sources for evaluation of greater sage-grouse (*Centrocercus urophasianus*) in response to precipitation, drought, and environmental factors within the Great Basin region of the USA, 1985–2021.

Category	Feature	Metrics	Analysis
Vegetation	Annual forb & grass[1]	a	i, ii, iii, iv
	Perennial forb & grass[1]	a	i, ii, iii, iv
	Bare ground[1]	a	i, ii, iii, iv
	Shrub[1]	a	i, ii, iii, iv
	Tree[1]	a	i, ii, iii, iv
	Sagebrush[13]	a	i, ii, iii, iv
	Non-sagebrush shrub[13]	a	i, ii, iii, iv
Topography	Elevation[14]	b	i, ii, iii, iv
	Heat load index[14]	c	i, ii, iii, iv
	Topographic roughness[14]	c	i, ii, iii, iv
	Topographic position[14]	c	i, ii, iii, iv
	Aspect[14]	c	i, ii, iii, iv
Mesic resources	Mesic rangeland[15]	d, e	i, ii, iii
	Wet meadow[15]	d, e	i, ii, iii
	Seasonal wetland[15]	d, e	i, ii, iii
	Riparian[15]	d, e	i, ii, iii
Anthropogenic	Percent developed imperviousness[5]	d	i, ii, iii, iv
Precipitation or drought	Precipitation[6]	f	i, ii, iii, iv
	Temperature[6]	f	i, ii, iii
	Standardized precipitation index[6]	f	i, ii, iii, iv
	Standardized precipitation evapotranspiration index[6]	f	i, ii, iii, iv
	Potential water deficit[6]	f	i, ii, iii, iv
	Vapor pressure deficit[6]	f	i, ii, iii, iv
	Snow water equivalent[6]	f	i, ii, iii, iv

a = mean % cover within circular neighborhood
b = average value within circular neighborhood
c = index value estimated within circular neighborhood
d = proportion of circular neighborhood occupied by feature
e = proximity to nearest feature, represented by exponential decay function
f = calculation of mean (temperature or index value) or cumulative (precipitation) over current or prior seasons

i = nest survival
ii = brood survival
iii = adult/yearling survival
iv = population state space model from lek count data

Radial buffers evaluated for circular neighborhoods:

- Nest survival: *75, 167, 260, 370, 439, 1,451 m*
- Brood, adult/yearling survival: *167, 260, 370, 439, 1,451 m*
- Population state space model from lek count data: *2.5, 5, 10 km*

Time periods considered for precipitation of drought covariates:

- Nest, brood, adult/yearling survival: *current month, current March-May, previous year, previous northern growing season, previous September-November, previous December-February*
- Population state space model from lek count data: *previous year, previous northern water year, previous northern growing season, previous December-February, previous March-May, previous June-August, previous September-November*

Sources:

Annual time series of percent cover, 1986 – present, version 3 from Rangeland Analysis Platform (https://rangelands.app/products/#cover)[1].

Annual time series of percent cover from rangeland fractional component (RCMAP) data products, 2023 release (https://www.mrlc.gov/data/type/rcmap-time-series-cover). Citation: Rigge, M. B., Bunde, B., Postma, K. & Shi, H. Rangeland Condition Monitoring Assessment and Projection (RCMAP) Fractional Component Time-Series Across the Western U.S. 1985-2023. *U.S. Geological Survey data release* (2024), https://doi.org/10.5066/P9SJXUI1[13].

Elevation or topographic indices derived from 30 m digital elevation model (DEM; https://www.usgs.gov/tools/national-map-viewer). Transformations and indices described in Evans, J. S., Oakleaf, J., Cushman, S. A. & Theobald, D. An ArcGIS Toolbox for Surface Gradient and Geomorphometric Modeling, version 2.0-0. http://evansmurphy.wixsite.com/evansspatial/arcgis-gradient-metrics-toolbox (2014)[14].

Availability and extent of different mesic resource types defined as areas with higher vegetation productivity during the late growing season (https://map.sagegrouseinitiative.com/ecosystem/mesic-resources/info?ll=43.4799,-110.7624&z=6) Citation: Donnelly, J. P., Naugle, D. E., Hagen, C. A. & Maestas, J. D. Public lands and private waters: scarce mesic resources structure land tenure and sage-grouse distributions. *Ecosphere* **7**, e01208 (2016) http://onlinelibrary.wiley.com/doi/10.1002/ecs2.1208/full[15] .

Urban impervious surfaces as percent of developed surface over every 30 m pixel, 2021 release (https://www.mrlc.gov/data/type/urban-imperviousness). Citation: Dewitz, J. A. National Land Cover Database (NLCD) 2019 Products (v2.0, June 2021). *U.S. Geological Survey data release* (2021) https://doi.org/10.5066/P9KZCM54[5].

Daily surface meteorological data at ~4 km spatial resolution covering the contiguous US from 1979 – present (https://www.climatologylab.org/gridmet.html). Citation: Abatzoglou, J. T. Development of gridded surface meteorological data for ecological applications and modelling. *International Journal of Climatology* **33**, 121–131 (2013)[6].

Literature cited

1. Allred, B. W. *et al.* Improving Landsat predictions of rangeland fractional cover with multitask learning and uncertainty. *Methods Ecol Evol* **12**, 841–849 (2021).

2. Rigge, M. *et al.* Quantifying western U.S. rangelands as fractional components with multi-resolution remote sensing and in situ data. *Remote Sens (Basel)* **12**, (2020).

3. Rigge, M. B., Bunde, B., Postma, K. & Shi, H. *Rangeland Condition Monitoring Assessment and Projection (RCMAP) Fractional Component Time-Series across the Western U.S. 1985-2021*. https://doi.org/10.5066/P9ODAZHC. (2022) doi:https://doi.org/10.5066/P9ODAZHC.

4. Donnelly, J. P. *et al.* Seasonal drought in North America's sagebrush biome structures dynamic mesic resources for sage-grouse. *Ecol Evol* **8**, 12492–12505 (2018).

5. Dewitz, J. A. *National Land Cover Database (NLCD) 2019 Products (v2.0, June 2021)*. (2021).

6. Abatzoglou, J. T. Development of gridded surface meteorological data for ecological applications and modelling. *International Journal of Climatology* **33**, 121–131 (2013).

7. Abatzoglou, J. T., McEvoy, D. J. & Redmond, K. T. The west wide drought tracker: Drought monitoring at fine spatial scales. *Bull Am Meteorol Soc* **98**, 1815–1820 (2017).

8. McGill, B. J. Matters of scale. *Science* vol. 328 575–576 Preprint at https://doi.org/10.1126/science.1188528 (2010).

9. Milligan, M. C. *et al.* Linking resource selection to population performance spatially to identify species' habitat across broad scales: An example of greater sage-grouse in a distinct population segment. *Ecol Evol* **14**, (2024).

10. Dudko, J. E., Coates, P. S. & Delehanty, D. J. Movements of female sage grouse Centrocercus urophasianus during incubation recess. *Ibis* **161**, 222–229 (2019).

11. Coates, P. S. *et al.* Evaluating greater sage-grouse seasonal space use relative to leks: Implications for surface use designations in sagebrush ecosystems. *J Wildl Manage* **77**, 1598–1609 (2013).

12. Doherty, K. E., Evans, J. S., Coates, P. S., Juliusson, L. M. & Fedy, B. C. Importance of regional variation in conservation planning: a rangewide example of the Greater Sage-Grouse. *Ecosphere* **7**, e01462 (2016).

13. Rigge, M. B., Bunde, B., Postma, K. & Shi, H. Rangeland Condition Monitoring Assessment and Projection (RCMAP) Fractional Component Time-Series Across the Western U.S. 1985-2023. *U.S. Geological Survey data release* (2024).

14. Evans, J. S., Oakleaf, J., Cushman, S. A. & Theobald, D. An ArcGIS Toolbox for Surface Gradient and Geomorphometric Modeling, version 2.0-0. http://evansmurphy.wixsite.com/evansspatial/arcgis-gradient-metrics-toolbox (2014).

15. Donnelly, J. P., Naugle, D. E., Hagen, C. A. & Maestas, J. D. Public lands and private waters: Scarce mesic resources structure land tenure and sage-grouse distributions. *Ecosphere* **7**, (2016).

APPENDIX S5

Bayesian latent scale and indicator variable selection

For each analysis (nest survival, brood survival, adult/yearling survival, population state space model), we considered multiple candidate scales for a suite of environmental covariates. Radial buffer distances were specified as 75, 167, 260, 370, 439, and 1,451 m for models of survival and 2.5, 5, and 10 km for population models (Appendix S4). If environmental features were continuous (e.g., % cover, elevation), we used a circular moving window to evaluate the mean value within the window across the different distances; if features were binary (e.g. presence/absence of mesic feature), we specified an exponential distance decay function and used the varying distances as the α in the function, exp($-d/\alpha$), which controls how rapidly a features influence decays with distance.

Considering multiple representations of the same feature at different spatial scales results in a large set of environmental covariates with potentially high pairwise correlations among the different representations. Due to the potential for collinearity and overfitting to complicate interpretation of modeling results, a model selection method was needed to 1) identify the scale with greatest support for the same feature, and 2) remove features that were not statistically supported as influential.

For all models, we used variable and scale selection methods within a Bayesian framework[1] to identify the environmental covariates with strongest influence on the various responses (nest survival, brood survival, adult/yearling survival, population growth rate). For survival models, we conducted a two-stage analysis to arrive at a final model for each response. First, we assigned covariates to groups, wherein members of the same group were known to be correlated (e.g., the same variable measured at multiple spatial scales or different time lags). We used latent indicator scale selection[2] to determine the most influential covariate within each group, by specifying a categorical prior distribution with uniform prior weights across categories. For example, the matrix of covariates **Z** includes groups $z' = z_1, z_2, ..., z_k$ (for k groups), and if each group comprises s scale (or lag) of measurement, then each $z_i \in \{z_{i1}, z_{i2}, ..., z_{is}\}$ with sub-indices representing group and scale. We specified a categorical distribution for each group, with prior weights equally balanced across each scale, $w_i = 1/s$, and $\sum_{s=1}^{s_i} w_{i,s} = 1$ [2]. At the group level, we imposed indicator variable selection[3,4] to assess evidence for inclusion of each covariate or proposed interaction term. This was done by specifying a dummy variable, $\omega_k \sim$ Bernoulli(0.5), such that its corresponding coefficient β_k was initially evaluated as $\omega_k \times \beta_k$ in each draw from the Markov chain Monte Carlo (MCMC) algorithm, and the proportion of the posterior distribution where $\omega_k = 1$ was interpreted as the inclusion probability for covariate k. Importantly, we constrained interpretation of inclusion probabilities to the proportion of the posterior distribution belonging to the highest probability scale or lag of measurement. For example, if group k had 3 candidate scales (s), and the 3rd scale had highest posterior probability, then we restricted the

estimate of inclusion probability based on ω_k where s=3. Once the first modeling stage was completed, we refit the model using only the representation or scale receiving greatest support within each group, and only if its posterior inclusion probability exceeded 0.5.

For population state-space models, we modified the model-fitting procedure to explore the relative influences of different precipitation and drought metrics across multiple time lags. We did this because we were less interested in identifying a 'best' model to explain sage-grouse population growth rates; rather, we sought to understand how effects of precipitation and drought might vary across seasons and cumulatively across prior years. Again, we used a two-stage analysis. In the first stage, we identified an appropriate scale for all non-climate covariates using latent indicator scale selection. Then we included each covariate at the scale receiving greatest support in a final set of models to test effects of precipitation and drought metrics. In this second stage, we fit 34 models independently to explore the influences of 5 metrics across 7 time-lags, excluding winter VPD because winter variation in VPD was negligible. We calculated WAIC (averaged across chains)[5] for each of these models to infer strength of support but focused our interpretation on the direction and strength of coefficients across metrics and time-lags.

Literature cited

1. Tenan, S., O'Hara, R. B., Hendriks, I. & Tavecchia, G. Bayesian model selection: the steepest mountain to climb. *Ecol Modell* **283**, 62–69 (2014).

2. Stuber, E. F., Gruber, L. F. & Fontaine, J. J. A Bayesian method for assessing multi-scale species-habitat relationships. *Landsc Ecol* **32**, 2365–2381 (2017).

3. Kuo, L. & Mallick, B. Variable selection for regression models. *Sankhyā: The Indian Journal of Statistics, Series B* 65–81 (1998).

4. O'Hara, R. B. & Sillanpää, M. J. A review of Bayesian variable selection methods: what, how and which. *Bayesian Anal* **4**, 85–117 (2009).

5. Vehtari, A., Gelman, A. & Gabry, J. Practical Bayesian model evaluation using leave-one-out cross-validation and WAIC. *Stat Comput* **27**, 1413–1432 (2017).

Appendix S6

Prior distributions

Specification of prior distributions for parameters to be estimated in hierarchical logistic exposure models of sage-grouse nest, brood, and adult survival from GPS & VHF telemetry data, and population state space models of sage-grouse lek count data.

Nest, brood, and adult logistic exposure survival models, stage I – variable and scale selection

$$\begin{aligned} &p_0 \sim Beta(1,1) \\ &\beta_0 = \text{logit}(p_0) \end{aligned} \quad (0.1)$$

$$\begin{aligned} &w_k \sim Bernoulli(0.5) \\ &\theta_k \sim Laplace(0, \lambda_b) \\ &\beta_k = w_k \times \theta_k \\ &\lambda_b \sim Unif(0.01, 10) \end{aligned} \quad (0.2)$$

$$\begin{aligned} &scale_{k_i} \sim Categorical(w_{k_i}) \\ &w_{k_i} = 1/s_i \\ &\sum_{s=1}^{s_i} w_{k_i} = 1 \end{aligned} \quad (0.3)$$

Nest, brood, and adult logistic exposure survival models, stage II – final model

$$\begin{aligned} &p_0 \sim Beta(1,1) \\ &\beta_0 = \text{logit}(p_0) \end{aligned} \quad (0.4)$$

$$\begin{aligned} &\beta_k \sim Laplace(0, \lambda_b) \\ &\lambda_b \sim Unif(0.01, 10) \end{aligned} \quad (0.5)$$

Population state space model stage I – variable and scale selection

$$\sigma_{lek} \sim Unif(0, 2) \quad (0.6)$$

$$n_{lek,t=1} \sim Unif(\log(2), \log(500)) \quad (0.7)$$

$$\begin{aligned} &\alpha_{clust} \sim N(\mu_{clust}, \sigma_{clust}) \\ &\mu_{clust} \sim N(0, 2) \\ &\sigma_{clust} \sim Unif(0, 10) \end{aligned} \quad (0.8)$$

$$
\begin{aligned}
&\mu_{\beta_j} \sim Laplace(0,\lambda) \\
&\sigma_{\beta_j} \sim Unif(0,10) \\
&\beta_{clust,j} \sim N(\mu_{\beta_j},\sigma_{\beta_j}) \\
&\lambda \sim Unif(0.1,10)
\end{aligned} \tag{0.9}
$$

$$
\begin{aligned}
&\mu_{\beta_{dd}} \sim N(0,1) \\
&\sigma_{\beta_{dd}} \sim Unif(0,10) \\
&\beta_{clust,dd} \sim N(\mu_{\beta_{dd}},\sigma_{\beta_{dd}})
\end{aligned} \tag{0.10}
$$

$$
\begin{aligned}
&scale_{j_i} \sim Categorical(w_{j_i}) \\
&w_{j_i} = 1/s_i \\
&\sum_{s=1}^{s_i} w_{j_i} = 1
\end{aligned} \tag{0.11}
$$

Population state space model stage II – final model

$$\sigma_{lek} \sim Unif(0,2) \tag{0.12}$$

$$n_{lek,t=1} \sim Unif(\log(2),\log(500)) \tag{0.13}$$

$$
\begin{aligned}
&\alpha_{clust} \sim N(\mu_{clust},\sigma_{clust}) \\
&\mu_{clust} \sim N(0,2) \\
&\sigma_{clust} \sim Unif(0,10)
\end{aligned} \tag{0.14}
$$

$$
\begin{aligned}
&\mu_{\beta_j} \sim Laplace(0,\lambda) \\
&\sigma_{\beta_j} \sim Unif(0,10) \\
&\beta_{clust,j} \sim N(\mu_{\beta_j},\sigma_{\beta_j}) \\
&\lambda \sim Unif(0.1,10)
\end{aligned} \tag{0.15}
$$

APPENDIX S7

Table S1. Rankings of 34 population state space models of Greater sage-grouse (*Centrocercus urophasianus*) lek counts in relation to precipitation and drought metrics, 1985–2021. Rankings were performed using the widely applicable information criterion (WAIC; also Watanabe-Akaike information criterion), while the log predictive posterior density (lppd) and estimate of effective number of parameters (pWAIC) are also listed. Each model was fit with a different precipitation or drought metric aggregated across one of seven specified time periods, while incorporating additional environmental covariates hypothesized to influence sage-grouse population dynamics (e.g., sagebrush, shrub cover, tree cover elevation, perennial grasses, annual grasses, impervious development; see main text). Time lags are also reported, which indicate the time span each metric represents relative to the current breeding season during which annual lek counts were conducted.

Metric	Time period	Time lag	WAIC	lppd	pWAIC
PPT	NGS	~ 6–12 months	143141	-60673	10898
SPEI	NWY	~ 6–18 months	143145	-60674	10899
PWD	JA	~ 6–9 months	143149	-60672	10902
VPD	NWY	~ 6–18 months	143151	-60672	10903
VPD	JA	~ 6–9 months	143160	-60674	10906
PWD	NWY	~ 6–18 months	143165	-60676	10907
PPT	NWY	~ 6–18 months	143169	-60675	10910
SPEI	LY	~ 0–12 months	143175	-60678	10909
SPEI	NGS	~ 6–12 months	143175	-60681	10907
SPI	NWY	~ 6–18 months	143178	-60680	10910
SPI	NGS	~ 6–12 months	143181	-60682	10908
VPD	NGS	~ 6–12 months	143181	-60678	10913
PPT	JA	~ 6–9 months	143183	-60679	10913
PPT	LY	~ 0–12 months	143184	-60673	10919
VPD	LY	~ 0–12 months	143184	-60675	10917
PWD	NGS	~ 6–12 months	143198	-60683	10916
PPT	DF	~ 0–3 months	143204	-60678	10924
SPI	LY	~ 0–12 months	143208	-60684	10920
SPEI	JA	~ 6–9 months	143208	-60683	10921
PWD	LY	~ 0–12 months	143209	-60680	10924
VPD	SN	~ 3–6 months	143218	-60678	10931
PWD	SN	~ 3–6 months	143219	-60683	10927
PWD	DF	~ 0–3 months	143236	-60685	10933
SPI	DF	~ 0–3 months	143237	-60686	10933
SPEI	MM	~ 9–12 months	143242	-60681	10940
VPD	MM	~ 9–12 months	143245	-60681	10941
SPEI	DF	~ 0–3 months	143249	-60684	10940
PWD	MM	~ 9–12 months	143249	-60682	10943
PPT	SN	~ 3–6 months	143253	-60691	10936
SPI	JA	~ 6–9 months	143265	-60688	10944
SPI	MM	~ 9–12 months	143265	-60687	10945
SPEI	SN	~ 3–6 months	143267	-60693	10941
PPT	MM	~ 9–12 months	143284	-60688	10954
SPI	SN	~ 3–6 months	143290	-60695	10950

Drought metric

PPT = cumulative precipitation
SPI = standardized precipitation index
SPEI = standardized precipitation evapotranspiration index
PWD = potential water deficit
VPD = vapor pressure deficit

Time period (see Appendix S4-Fig. S1)
MM = March–May
JA = July–August
SN = September–November
DF = December–February
NGS = Northern growing season (Oct – Sep)
LY = Last year (Mar – Mar)
NWY = Northern water year (Oct – Sep)

APPENDIX S8

Table S1

Table S1. Bayesian latent indicator scale and variable selection results from a hierarchical logistic exposure model of greater sage-grouse (*Centrocercus urophasianus*) nest survival estimated from data collected in the Great Basin region of the USA, 2003–2021. Groups were defined by either the same source variable measured at multiple spatial scales, or variables that were highly correlated measured at multiple scales. Group probabilities represent the posterior probability of a variable being selected from the larger set of candidate variables, whereas inclusion probability represents the posterior probability of variable inclusion in the top model. The β value represents the coefficient midpoint of the posterior distribution, along with its 2.5th and 97.5th percentiles. Variables selected for the final model stage are outlined and highlighted in forest green.

Group	Variable	Group probability	Inclusion probability	β	2.5th	97.5th
Sagebrush	% Cover 75m	0.273	0.680	0.053	0.000	0.155
	% Cover 167m	0.181				
	% Cover 260m	0.151				
	% Cover 370m	0.138				
	% Cover 439m	0.133				
	% Cover 1451m	0.124				
Annual forb	% Cover 75m	0.246	0.754	-0.073	-0.196	0.000
& grass	% Cover 167m	0.235				
	% Cover 260m	0.156				
	% Cover 370m	0.137				
	% Cover 439m	0.135				
	% Cover 1451m	0.092				
Perennial forb	% Cover 75m	0.160				
& grass	% Cover 167m	0.177	0.315	0.007	-0.041	0.080
	% Cover 260m	0.171				
	% Cover 370m	0.163				
	% Cover 439m	0.166				
	% Cover 1451m	0.162				
Bare ground	% Cover 75m	0.176				
	% Cover 167m	0.195	0.639	-0.054	-0.183	0.006
	% Cover 260m	0.168				
	% Cover 370m	0.153				
	% Cover 439m	0.149				
	% Cover 1451m	0.160				
Tree	% Cover 75m	0.164				
	% Cover 167m	0.164				
	% Cover 260m	0.171				
	% Cover 370m	0.162				
	% Cover 439m	0.168				

	% Cover 1451m	0.172	0.287	-0.005	-0.033	0.076
Non-sagebrush shrub	% Cover 75m	0.053				
	% Cover 167m	0.146				
	% Cover 260m	0.100				
	% Cover 370m	0.077				
	% Cover 439m	0.080				
	% Cover 1451m	0.545	0.998	0.201	0.092	0.309
Elevation	Mean 75m	0.162				
	Mean 167m	0.168				
	Mean 260m	0.167				
	Mean 370m	0.164				
	Mean 439m	0.176	0.358	0.012	-0.044	0.116
	Mean 1451m	0.163				
Topographic roughness	Mean 75m	0.115				
	Mean 167m	0.134				
	Mean 260m	0.146				
	Mean 370m	0.216				
	Mean 439m	0.276	0.695	0.056	0.000	0.158
	Mean 1451m	0.113				
Topographic position	Mean 75m	0.152				
	Mean 167m	0.161				
	Mean 260m	0.161				
	Mean 370m	0.180				
	Mean 439m	0.185	0.336	-0.012	-0.081	0.010
	Mean 1451m	0.159				
Aspect & heat load	Transformed aspect, mean 75m	0.087				
	Transformed aspect, mean 167m	0.087				
	Transformed aspect, mean 260m	0.082				
	Transformed aspect, mean 370m	0.080				
	Transformed aspect, mean 439m	0.078				
	Transformed aspect, mean 1451m	0.103	0.412	-0.018	-0.093	0.008
	Heat load index, mean 75m	0.082				
	Heat load index, mean 167m	0.081				
	Heat load index, mean 260m	0.083				
	Heat load index, mean 370m	0.083				
	Heat load index, mean 439m	0.079				
	Heat load index, mean 1451m	0.075				
Mesic rangeland	Proximity, α = 75m	0.149				
	Proximity, α = 167m	0.162				
	Proximity, α = 260m	0.171				
	Proximity, α = 370m	0.174				
	Proximity, α = 439m	0.170				

	Proximity, α = 1451m	0.175	0.376	-0.016	-0.102	0.012
Seasonal wetland	Proximity, α = 75m	0.158				
	Proximity, α = 167m	0.171				
	Proximity, α = 260m	0.162				
	Proximity, α = 370m	0.164				
	Proximity, α = 439m	0.164				
	Proximity, α = 1451m	0.181	0.370	0.015	-0.002	0.091
Wet meadow	Proximity, α = 75m	0.161				
	Proximity, α = 167m	0.155				
	Proximity, α = 260m	0.158				
	Proximity, α = 370m	0.169				
	Proximity, α = 439m	0.171				
	Proximity, α = 1451m	0.186	0.341	-0.012	-0.088	0.012
Riparian	Proximity, α = 75m	0.166				
	Proximity, α = 167m	0.163				
	Proximity, α = 260m	0.170				
	Proximity, α = 370m	0.178	0.248	-0.003	-0.051	0.033
	Proximity, α = 439m	0.163				
	Proximity, α = 1451m	0.160				
Temperature	Mean Tmax	0.130				
	Maximum Tmax	0.136				
	Mean Tmin	0.256				
	Minimum Tmin	0.142				
	Vapor pressure deficit	0.336	0.712	-0.058	-0.161	0.000
Snow water equivalent	Mean spring SWE	0.497				
	Max spring SWE	0.503	0.294	0.005	-0.036	0.078
Precipitation	PPT	0.164				
	SPI	0.160				
	SPEI	0.168				
	PPT, March-May	0.196	0.260	0.001	-0.042	0.054
	SPI, March-May	0.147				
	SPEI, March-May	0.165				
Lagged Precipitation	PPT, previous year	0.058				
	PWD, previous year	0.065				
	SPI, previous year	0.058				
	SPEI, previous year	0.069				
	PPT, previous growing season	0.055				
	PWD, previous growing season	0.069				
	SPI, previous growing season	0.062				
	SPEI, previous growing season	0.058				
	PPT, previous Sept-Nov	0.053				
	PWD, previous Sept-Nov	0.059				

	SPI, previous Sept-Nov	0.054				
	SPEI, previous Sept-Nov	0.063				
	PPT, previous Dec-Feb	0.066				
	PWD, previous Dec-Feb	0.064				
	SPI, previous Dec-Feb	0.072				
	SPEI, previous Dec-Feb	0.075	0.224	0.002	-0.039	0.057
Temperature × Day of season	Mean Tmin					
	Minimum Tmin					
	Mean Tmax					
	Maximum Tmax					
	Vapor pressure deficit		0.250	-0.001	-0.049	0.041
Snow water equivalent × Day of season	Mean spring SWE					
	Max spring SWE		0.322	0.011	-0.018	0.093
Precipitation × Day of season	PPT					
	SPI					
	SPEI					
	PPT, March-May		0.243	0.001	-0.038	0.048
	SPI, March-May					
	SPEI, March-May					
Lagged precipitation × Day of season	PPT, previous year					
	PWD, previous year					
	SPI, previous year					
	SPEI, previous year		0.326	-0.013	-0.092	0.028
	PPT, previous growing season					
	PWD, previous growing season					
	SPI, previous growing season					
	SPEI, previous growing season					
	PPT, previous Sept-Nov					
	PWD, previous Sept-Nov					
	SPI, previous Sept-Nov					
	SPEI, previous Sept-Nov					
	PPT, previous Dec-Feb					
	PWD, previous Dec-Feb					
	SPI, previous Dec-Feb					
	SPEI, previous Dec-Feb					
Individual effects	Female age		0.340	0.006	-0.067	0.106
	Day of year		0.984	0.134	0.030	0.233

Table S2

Table S2. Bayesian latent indicator scale and variable selection results from a hierarchical logistic exposure model of greater sage-grouse (*Centrocercus urophasianus*) brood survival estimated from data

collected in the Great Basin region of the USA, 2003–2021. Groups were defined by either the same source variable measured at multiple spatial scales, or variables that were highly correlated measured at multiple scales. Group probabilities represent the posterior probability of a variable being selected from the larger set of candidate variables, whereas inclusion probability represents the posterior probability of variable inclusion in the top model. The β value represents the coefficient midpoint of the posterior distribution, along with its 2.5th and 97.5th percentiles. Variables selected for the final model stage are outlined and highlighted in forest green.

Group	Variable	Group probability	Inclusion probability	β	2.5th	97.5th
Sagebrush	% Cover 167m	0.209				
	% Cover 260m	0.222				
	% Cover 370m	0.251	0.355*	0.009	-0.050	0.104
	% Cover 439m	0.233				
	% Cover 1451m	0.085				
Non-sagebrush shrub	% Cover 167m	0.184				
	% Cover 260m	0.176				
	% Cover 370m	0.171				
	% Cover 439m	0.185				
	% Cover 1451m	0.284	0.568	0.050	-0.025	0.219
Annual forb & grass	% Cover 167m	0.198				
	% Cover 260m	0.192				
	% Cover 370m	0.200				
	% Cover 439m	0.206	0.337	0.006	-0.066	0.106
	% Cover 1451m	0.204				
Perennial grass	% Cover 167m	0.194				
	% Cover 260m	0.193				
	% Cover 370m	0.189				
	% Cover 439m	0.199				
	% Cover 1451m	0.224	0.398	0.016	-0.046	0.137
Bare ground	% Cover 167m	0.126				
	% Cover 260m	0.113				
	% Cover 370m	0.135				
	% Cover 439m	0.147				
	% Cover 1451m	0.479	0.830	-0.099	-0.230	0.000
Tree	% Cover 167m	0.171				
	% Cover 260m	0.190				
	% Cover 370m	0.197				
	% Cover 439m	0.190				
	% Cover 1451m	0.252	0.526	0.037	-0.012	0.168
Elevation	Mean 167m	0.189				
	Mean 260m	0.197				
	Mean 370m	0.213	0.332	-0.003	-0.099	0.080
	Mean 439m	0.205				

	Mean 1451m	0.195				
Topographic roughness	Mean 167m	0.331	0.624	-0.049	-0.169	0.010
	Mean 260m	0.205				
	Mean 370m	0.177				
	Mean 439m	0.145				
	Mean 1451m	0.142				
Topographic position	Mean 167m	0.201				
	Mean 260m	0.207	0.336	-0.011	-0.094	0.030
	Mean 370m	0.198				
	Mean 439m	0.202				
	Mean 1451m	0.191				
Aspect & heat load	Transformed aspect, mean 167m	0.109				
	Transformed aspect, mean 260m	0.110				
	Transformed aspect, mean 370m	0.100				
	Transformed aspect, mean 439m	0.103				
	Transformed aspect, mean 1451m	0.160	0.564	0.042	-0.013	0.170
	Heat load index, mean 167m	0.084				
	Heat load index, mean 260m	0.076				
	Heat load index, mean 370m	0.078				
	Heat load index, mean 439m	0.080				
	Heat load index, mean 1451m	0.099				
Mesic rangeland	Proximity, α = 167m	0.211				
	Proximity, α = 260m	0.215	0.454	0.030	-0.023	0.176
	Proximity, α = 370m	0.197				
	Proximity, α = 439m	0.197				
	Proximity, α = 1451m	0.180				
Seasonal wetland	Proximity, α = 167m	0.219	0.380*	0.017	-0.043	0.155
	Proximity, α = 260m	0.218				
	Proximity, α = 370m	0.203				
	Proximity, α = 439m	0.193				
	Proximity, α = 1451m	0.168				
Wet meadow	Proximity, α = 167m	0.206				
	Proximity, α = 260m	0.202				
	Proximity, α = 370m	0.195				
	Proximity, α = 439m	0.208	0.366	-0.011	-0.112	0.042
	Proximity, α = 1451m	0.189				
Riparian	Proximity, α = 167m	0.193				
	Proximity, α = 260m	0.195				
	Proximity, α = 370m	0.194				
	Proximity, α = 439m	0.198				
	Proximity, α = 1451m	0.220	0.330	0.009	-0.041	0.104
Temperature	Mean Tmin	0.369	0.384*	0.010	-0.065	0.115

	Minimum Tmin	0.353				
	Mean Tmax	0.106				
	Maximum Tmax	0.083				
	Vapor pressure deficit	0.088				
Snow water equivalent	Mean spring SWE	0.505	0.392	0.016	-0.035	0.131
	Max spring SWE	0.495				
Precipitation	PPT	0.127				
	SPI	0.072				
	SPEI	0.048				
	PPT, March-May	0.244				
	SPI, March-May	0.370	0.860	-0.115	-0.253	0.000
	SPEI, March-May	0.140				
Lagged precipitation	PPT, previous year	0.032				
	PWD, last year	0.047				
	SPI, last year	0.156				
	SPEI, last year	0.075				
	PPT, northern growing season	0.074				
	PWD, northern growing season	0.018				
	SPI, northern growing season	0.023				
	SPEI, northern growing season	0.019				
	PPT, Sept-Nov	0.088				
	PWD, Sept-Nov	0.038				
	SPI, Sept-Nov	0.167	0.412	0.019	-0.043	0.146
	SPEI, Sept-Nov	0.031				
	PPT, Dec-Feb	0.024				
	PWD, Dec-Feb	0.031				
	SPI, Dec-Feb	0.095				
	SPEI, Dec-Feb	0.083				
	Day of year		0.652	0.067	-0.017	0.233
	Hen age		0.637	0.085	-0.032	0.342
	Brood age		0.572	0.049	-0.023	0.205
	Brood age*Day of year		0.376	0.011	-0.062	0.137
Sagebrush × Brood age	% Cover 167m					
	% Cover 260m					
	% Cover 370m		0.811	-0.104	-0.249	0.000
	% Cover 439m					
	% Cover 1451m					
Non-sagebrush shrub × Brood age	% Cover 167m					
	% Cover 260m					
	% Cover 370m					
	% Cover 439m					
	% Cover 1451m		0.393	-0.010	-0.117	0.062

Annual grass × Brood age	% Cover 167m				
	% Cover 260m				
	% Cover 370m				
	% Cover 439m	0.389	0.015	-0.043	0.136
	% Cover 1451m				
Perennial grass × Brood age	% Cover 167m				
	% Cover 260m				
	% Cover 370m				
	% Cover 439m				
	% Cover 1451m	0.398	0.016	-0.041	0.129
Bare ground × Brood age	% Cover 167m				
	% Cover 260m				
	% Cover 370m				
	% Cover 439m				
	% Cover 1451m	0.510	-0.035	-0.167	0.021
Tree × Brood age	% Cover 167m				
	% Cover 260m				
	% Cover 370m				
	% Cover 439m				
	% Cover 1451m	0.385	-0.015	-0.116	0.028
Elevation × Brood age	Mean 167m				
	Mean 260m				
	Mean 370m	0.353	0.001	-0.087	0.091
	Mean 439m				
	Mean 1451m				
Topographic roughness × Brood age	Mean 167m	0.415	0.020	-0.017	0.125
	Mean 260m				
	Mean 370m				
	Mean 439m				
	Mean 1451m				
Topographic position × Brood age	Mean 167m				
	Mean 260m	0.373	0.013	-0.028	0.099
	Mean 370m				
	Mean 439m				
	Mean 1451m				
Aspect or heat load × Brood age	Transformed aspect, mean 167m				
	Transformed aspect, mean 260m				
	Transformed aspect, mean 370m				
	Transformed aspect, mean 439m				
	Transformed aspect, mean 1451m	0.458	-0.026	-0.140	0.024
	Heat load index, mean 167m				
	Heat load index, mean 260m				

	Heat load index, mean 370m				
	Heat load index, mean 439m				
	Heat load index, mean 1451m				
Mesic rangeland × Brood age	Proximity, α = 167m				
	Proximity, α = 260m	0.367	0.012	-0.048	0.114
	Proximity, α = 370m				
	Proximity, α = 439m				
	Proximity, α = 1451m				
Seasonal wetland × Brood age	Proximity, α = 167m	0.503	-0.034	-0.162	0.020
	Proximity, α = 260m				
	Proximity, α = 370m				
	Proximity, α = 439m				
	Proximity, α = 1451m				
Wet meadow × Brood age	Proximity, α = 167m				
	Proximity, α = 260m				
	Proximity, α = 370m				
	Proximity, α = 439m	0.368	0.011	-0.042	0.119
	Proximity, α = 1451m				
Riparian × Brood age	Proximity, α = 167m				
	Proximity, α = 260m				
	Proximity, α = 370m				
	Proximity, α = 439m				
	Proximity, α = 1451m	0.458	0.023	-0.027	0.139
Temperature × Brood age	Mean Tmin	0.786	-0.103	-0.268	0.000
	Minimum Tmin				
	Mean Tmax				
	Maximum Tmax				
	Vapor pressure deficit				
Snow water equivalent × Brood age	Mean spring SWE	0.325	0.000	-0.076	0.075
	Max spring SWE				
Precipitation × Brood age	PPT				
	SPI				
	SPEI				
	PPT, March-May				
	SPI, March-May	0.378	0.010	-0.058	0.122
	SPEI, March-May				
Lagged precipitation × Brood age	PPT, last year				
	PWD, last year				
	SPI, last year				
	SPEI, last year				
	PPT, northern growing season				

	PWD, northern growing season				
	SPI, northern growing season				
	SPEI, northern growing season				
	PPT, Sept-Nov				
	PWD, Sept-Nov				
	SPI, Sept-Nov	0.394	-0.013	-0.131	0.057
	SPEI, Sept-Nov				
	PPT, Dec-Feb				
	PWD, Dec-Feb				
	SPI, Dec-Feb				
	SPEI, Dec-Feb				
Temperature × Day of season	Mean Tmin	0.422	-0.019	-0.148	0.048
	Minimum Tmin				
	Mean Tmax				
	Maximum Tmax				
	Vapor pressure deficit				
Snow water equivalent × Day of season	Mean spring SWE	0.354	0.003	-0.074	0.096
	Max spring SWE				
Precipitation × Day of season	PPT				
	SPI				
	SPEI				
	PPT, March-May				
	SPI, March-May	0.467	0.029	-0.043	0.180
	SPEI, March-May				
Lagged precipitation × Day of season	PPT, last year				
	PWD, last year				
	SPI, last year				
	SPEI, last year				
	PPT, northern growing season				
	PWD, northern growing season				
	SPI, northern growing season				
	SPEI, northern growing season				
	PPT, Sept-Nov				
	PWD, Sept-Nov				
	SPI, Sept-Nov	0.911	-0.141	-0.285	0.000
	SPEI, Sept-Nov				
	PPT, Dec-Feb				
	PWD, Dec-Feb				
	SPI, Dec-Feb				
	SPEI, Dec-Feb				
Individual effects	Day of year	0.652	0.067	-0.017	0.233

	Female age		0.637	0.085	-0.032	0.342
	Brood age		0.572	0.049	-0.023	0.205
	Brood age × Day of season		0.376	0.011	-0.062	0.137

*Implies that the metric was selected for inclusion in second stage of based on support for an interaction effect with another metric, rather than the main effect itself. However, the main effect is included in this interaction in the second modeling stage.

Table S3

Table S3. Bayesian latent indicator scale and variable selection results from a hierarchical logistic exposure model of greater sage-grouse (*Centrocercus urophasianus*) adult and yearling survival estimated from data collected in the Great Basin region of the USA, 2003–2021. Groups were defined by either the same source variable measured at multiple spatial scales, or variables that were highly correlated measured at multiple scales. Group probabilities represent the posterior probability of a variable being selected from the larger set of candidate variables, whereas inclusion probability represents the posterior probability of variable inclusion in the top model. The β value represents the coefficient midpoint of the posterior distribution, along with its 2.5th and 97.5th percentiles. Variables selected for the final model stage are outlined and highlighted in forest green.

Group	Variable	Group probability	Inclusion probability	β	2.5th	97.5th
Sagebrush	% Cover 167m	0.192				
	% Cover 260m	0.194				
	% Cover 370m	0.194				
	% Cover 439m	0.194				
	% Cover 1451m	0.226	0.426	-0.022	-0.131	0.021
Non-sagebrush shrub	% Cover 167m	0.198				
	% Cover 260m	0.169				
	% Cover 370m	0.165				
	% Cover 439m	0.166				
	% Cover 1451m	0.302	0.674	-0.063	-0.199	0.000
Annual forb & grass	% Cover 167m	0.198				
	% Cover 260m	0.197				
	% Cover 370m	0.198				
	% Cover 439m	0.211	0.369	-0.011	-0.114	0.045
	% Cover 1451m	0.197				
Perennial forb & grass	% Cover 167m	0.378	0.822	0.134	0.000	0.313
	% Cover 260m	0.209				
	% Cover 370m	0.170				
	% Cover 439m	0.144				
	% Cover 1451m	0.099				
Bare ground	% Cover 167m	0.218				
	% Cover 260m	0.220				
	% Cover 370m	0.226	0.723	0.085	-0.002	0.234
	% Cover 439m	0.202				

	% Cover 1451m	0.134				
Tree	% Cover 167m	0.324	0.753	-0.048	-0.117	0.000
	% Cover 260m	0.190				
	% Cover 370m	0.145				
	% Cover 439m	0.163				
	% Cover 1451m	0.177				
Impervious developed	% Cover 167m	0.189				
	% Cover 260m	0.244	0.432	-0.021	-0.097	0.002
	% Cover 370m	0.201				
	% Cover 439m	0.182				
	% Cover 1451m	0.183				
Elevation	Mean 167m	0.195				
	Mean 260m	0.195				
	Mean 370m	0.189				
	Mean 439m	0.203				
	Mean 1451m	0.219	0.493	-0.038	-0.206	0.028
Topographic roughness	Mean 167m	0.189				
	Mean 260m	0.194				
	Mean 370m	0.200				
	Mean 439m	0.205				
	Mean 1451m	0.212	0.345	-0.013	-0.102	0.018
Topographic position	Mean 167m	0.087				
	Mean 260m	0.565	1.000	-0.163	-0.231	-0.091
	Mean 370m	0.233				
	Mean 439m	0.115				
	Mean 1451m	0.000				
Aspect & heat load	Transformed aspect, mean 167m	0.092				
	Transformed aspect, mean 260m	0.102				
	Transformed aspect, mean 370m	0.111	0.352	-0.015	-0.100	0.017
	Transformed aspect, mean 439m	0.101				
	Transformed aspect, mean 1451m	0.090				
	Heat load index, mean 167m	0.094				
	Heat load index, mean 260m	0.101				
	Heat load index, mean 370m	0.099				
	Heat load index, mean 439m	0.109				
	Heat load index, mean 1451m	0.101				
Mesic rangeland	Proximity, α = 167m	0.189				
	Proximity, α = 260m	0.222				
	Proximity, α = 370m	0.220				
	Proximity, α = 439m	0.238	0.731	-0.075	-0.209	0.000
	Proximity, α = 1451m	0.131				
Seasonal wetland	Proximity, α = 167m	0.194				

	Proximity, α = 260m	0.200				
	Proximity, α = 370m	0.207				
	Proximity, α = 439m	0.193				
	Proximity, α = 1451m	0.207	0.256	-0.005	-0.068	0.036
Wet meadow	Proximity, α = 167m	0.145				
	Proximity, α = 260m	0.141				
	Proximity, α = 370m	0.142				
	Proximity, α = 439m	0.143				
	Proximity, α = 1451m	0.429	0.801	0.091	0.000	0.208
Riparian	Proximity, α = 167m	0.188				
	Proximity, α = 260m	0.196				
	Proximity, α = 370m	0.202				
	Proximity, α = 439m	0.218	0.331	-0.010	-0.081	0.021
	Proximity, α = 1451m	0.195				
Temperature	Mean Tmin	0.020				
	Minimum Tmin	0.021				
	Mean Tmax	0.353				
	Maximum Tmax	0.035				
	Vapor pressure deficit	0.571	0.984	0.302	0.056	0.457
Snow water equivalent	Mean spring SWE	0.553	0.864	-0.086	-0.169	0.000
	Max spring SWE	0.447				
Precipitation	PPT	0.143				
	SPI	0.160				
	SPEI	0.184				
	PPT, March-May	0.139				
	SPI, March-May	0.195	0.311	0.008	-0.035	0.089
	SPEI, March-May	0.179				
Lagged precipitation	PPT, last year	0.001				
	PWD, last year	0.000				
	SPI, last year	0.001				
	SPEI, last year	0.000				
	PPT, northern growing season	0.077				
	PWD, northern growing season	0.162				
	SPI, northern growing season	0.182				
	SPEI, northern growing season	0.529	0.999	0.17	0.087	0.248
	PPT, Sept-Nov	0.001				
	PWD, Sept-Nov	0.032				
	SPI, Sept-Nov	0.004				
	SPEI, Sept-Nov	0.008				
	PPT, Dec-Feb	0.000				
	PWD, Dec-Feb	0.000				
	SPI, Dec-Feb	0.000				

	SPEI, Dec-Feb	0.002				
Temperature × Day of season	Mean Tmin					
	Minimum Tmin					
	Mean Tmax					
	Maximum Tmax					
	Vapor pressure deficit		0.407	0.022	-0.022	0.143
Snow water equivalent × Day of season	Mean spring SWE		0.219	0.001	-0.037	0.040
	Max spring SWE					
Precipitation × Day of season	PPT					
	SPI					
	SPEI					
	PPT, March-May					
	SPI, March-May		0.42	0.021	-0.014	0.114
	SPEI, March-May					
Lagged precipitation × Day of season	PPT, last year					
	PWD, last year					
	SPI, last year					
	SPEI, last year					
	PPT, northern growing season					
	PWD, northern growing season					
	SPI, northern growing season					
	SPEI, northern growing season					
	PPT, Sept-Nov					
	PWD, Sept-Nov					
	SPI, Sept-Nov		0.923	-0.006	-0.079	0.033
	SPEI, Sept-Nov					
	PPT, Dec-Feb					
	PWD, Dec-Feb					
	SPI, Dec-Feb					
	SPEI, Dec-Feb					
Individual effects	Day of year		0.521	0.05	-0.057	0.304
	Sex		0.507	0.041	-0.065	0.255
	GPS		0.415	0.022	-0.063	0.192
	Female age		0.341	0.001	-0.096	0.107
	Translocated		0.469	-0.014	-0.26	0.184

APPENDIX S9

Explanation and interpretation of modeled landcover composition effects on sage-grouse vital rates (nest, brood, and adult survival) and apparent population growth (λ)

Many previous studies have investigated the effects of landcover composition on individual sage-grouse demographic rates[1–3], but few have comprehensively demonstrated landcover effects on population growth rates at broad spatial scales (but see Coates et al.[4], Monroe et al.[5], Prochazka et al.[6]) while also capturing effects driven by climate and weather. Our results indicate that sage-grouse population growth rates were positively associated with sagebrush cover, likely reflecting the importance of sagebrush for sage-grouse nest concealment[1,7,8]. Sage-grouse population trends in Wyoming were positively associated with sagebrush cover at approximately the same scale we found (5 km)[5], and the magnitude of the effect we detected was very similar to that reported in Nevada[4]. While non-sagebrush shrub cover was positively associated with nest survival, there was some evidence that older brood and adult survival declined with increasing non-sagebrush shrub cover. Some non-sagebrush shrub species (e.g., antelope bitterbrush; *Purshia tridentata*) are often used for nest sites and contribute to overall nest cover [9,10]. However, species composition of the non-sagebrush shrub component likely varies seasonally and across life stages and may include shrub species that are associated with marginal, early successional, or degraded sage-grouse habitat[9,11] outside of the nesting season.

Population growth was negatively influenced by tree cover, perhaps through the negative effects of trees on adult survival[12,13]. Higher tree cover within conifer-dominated landcover types was associated with lower sage-grouse carrying capacity in Wyoming[14], no leks remained active once conifer cover exceeded 4% in Oregon[15], and sage-grouse population growth rates on conifer-removal sites increased relative to control sites in the northern Great Basin[16]. Our results suggested a weak positive association between overall tree cover and brood survival, which in part may reflect the co-occurrence of trees, higher elevation, and higher productivity mesic sites that provide critical forage resources for growing broods[17,18]. Such high-elevation areas may be of greater importance in drier years, when food resources for broods are limited in less-risky lower elevation areas with fewer trees[19,20]. In addition, our analysis was unable to isolate conifers from other tree species. The negative effects of tree cover on sage-grouse populations are typically assumed to be associated with the encroachment of early successional conifers into sagebrush, but some wooded areas (e.g., aspen edges) may in some cases be beneficial to sage-grouse, again given their proximity to food-rich higher elevation habitats. Disentangling these potential effects was beyond the scope of our analysis, though higher resolution landcover products could help identify contrasting effects of conifers and other tree species on multiple sage-grouse life stages. Yet, because the net effect of tree cover on population growth was negative, it is likely that our models of adult, brood, and nest survival did not comprehensively explain all pathways through which conifer and/or total tree cover can contribute to reduced population performance.

Our analysis largely corroborated previous findings, that proximity to mesic habitats improve brood survival[18,21] and influence overall sage-grouse population dynamics[17,22], but provided additional evidence that such effects may be complex and context-dependent[19,23]. Brood survival declined with increasing distance to mesic rangelands, which may remain productive later into the summer during high precipitation years, but the strength of such effects might depend on the availability of other mesic resource types in low precipitation years[17]. For example, we found that the survival of early-aged broods also increased with proximity to seasonal wetlands and that adult survival increased with greater proximity to wet meadows. Donnelly et al.[17] speculated that a diversity of complementary mesic resources may be required for the long-term persistence of sage-grouse populations, and that drought-resilient wet meadows support adult survival during dry periods while temporally dynamic mesic rangelands support pulses of recruitment during wet periods. However, we found that late brood survival was negatively associated with proximity to seasonal wetlands. Small scale and isolated point water sources may attract predators, subject sage-grouse to disturbance by livestock and feral equids[4,20], or limit survival through other density-dependent effects[20]. Further, sage-grouse broods might face tradeoffs between foraging and predator avoidance along the ecotone between wetlands and uplands, and those tradeoffs might depend on current-year conditions and overall landscape context[23]. For example, selection of edge habitats might balance the need for chicks to obtain key forage resources without straying far from protective shrub cover (Aldridge & Boyce, 2007). Consistent with these ideas, the availability of meadow edge was more influential to brood survival than was meadow size in the southwestern Great Basin[24], likely because meadow interiors are associated with lower cover and greater predation risk[23,25]. The proximity and configuration of productive wet meadow habitat, relative to uplands with adequate protective cover, may vary among wet and dry years [17,23], however we were unable to model mesic resource availability as a time-varying effect. Hence, additional research could help clarify nuances related to the relative importance of different types of mesic resources, patch size and composition of mesic habitat, critical thresholds in indices of productivity (e.g., NDVI)[22], and how these relationships vary among life-stages and are mediated by interactions with predation and landscape context.

The strongest landcover effect we detected on sage-grouse population growth rates was the negative effect of annual forb and grass cover, corroborating the results of a recent range-wide analysis[6]. Sage-grouse population growth rates in the Great Basin have also been shown to decline in response to greater cumulative burned area, which is strongly correlated with the expansion of invasive annual grasses and associated changes in the structure and function of sagebrush ecosystems[26,27]. Annual grasses outcompete and displace native vegetation, increase the continuity of fine fuels, and drive an intensifying positive feedback cycle with wildfire[28,29]. Sage-grouse's high site fidelity to what were once stable sagebrush habitats[30] leads them to continue nesting in recently burned areas[26] where they experience acute and longer-term reductions in nest, chick, and adult survival[31,32]. Annual grasses may also dampen the association

of sage-grouse population growth with precipitation and limit the ability of populations to respond to years of favorable weather[3,27].

Sage-grouse population growth rates declined with increasing impervious development. Impervious surfaces are associated with urban development, anthropogenic infrastructure, roads, and unreclaimed mine sites[33,34] and often considered an indicator of habitat fragmentation[35]. In combination with anthropogenic food subsidies (e.g., roads, rest areas, and landfills), impervious surfaces in the Great Basin were associated with increased occurrence of common ravens, a primary sage-grouse nest predator[36]. Sage-grouse may also behaviorally avoid the noise, light, and human activity associated with urban and industrial (e.g., mining, energy) development[37] which could contribute to local extirpation of leks[38,39]. Finally, impervious development may increase runoff, reduce water infiltration, and limit groundwater recharge that supports herbaceous productivity[33], thereby reducing sage-grouse habitat suitability.

Literature cited

1. Lockyer, Z. B., Coates, P. S., Casazza, M. L., Espinosa, S. & Delehanty, D. J. Nest-site selection and reproductive success of greater sage-grouse in a fire-affected habitat of northwestern Nevada. *J Wildl Manage* **79**, 785–797 (2015).

2. Severson, J. P. *et al.* Better living through conifer removal: A demographic analysis of sage-grouse vital rates. *PLoS One* **12**, (2017).

3. Blomberg, E. J., Sedinger, J. S., Atamian, M. T. & Nonne, D. V. Characteristics of climate and landscape disturbance influence the dynamics of greater sage-grouse populations. *Ecosphere* **3**, 1–20 (2012).

4. Coates, P. S., O'Neil, S. T., Munoz, D. A., Dwight, I. A. & Tull, J. C. Sage-grouse population dynamics are adversely affected by overabundant feral horses. *Journal of Wildlife Management* (2021) doi:10.1002/jwmg.22089.

5. Monroe, A. P. *et al.* Spatial scale selection for informing species conservation in a changing landscape. *Ecosphere* **13**, (2022).

6. Prochazka, B. G. *et al.* Evaluating the Sagebrush Conservation Design Strategy Through the Performance of a Sagebrush Indicator Species. *Rangel Ecol Manag* **97**, 146–159 (2024).

7. Hansen, C. P. *et al.* Microsite selection and survival of greater sage-grouse nests in south-central Wyoming. *Journal of Wildlife Management* **80**, 862–876 (2016).

8. Kaczor, N. W. *et al.* Nesting success and resource selection of greater sage-grouse. in *Studies in Avian Biology 39: Ecology, conservation, and management of Grouse* 107–118 (2011).

9. Connelly, J. W., Rinkes, E. T. & Braun, C. E. Characteristics of greater sage-grouse habitats: a landscape species at micro- and macroscales. in *Studies in Avian Biology: Greater sage-grouse: ecology and conservation of a landscape species and its habitats* (eds. Knick, S. T. & Connelly, J. W.) vol. 38 69–84 (University of California Press, Berkeley, 2011).

10. Aldridge, C. L. Identifying habitats for persistence of greater sage-grouse (Centrocercus urophasianus) in Alberta, Canada. (University of Alberta, Edmonton, 2005).

11. Orning, E. K., Heinrichs, J. A., Pyke, D. A., Coates, P. S. & Aldridge, C. L. Using state-and-transition simulation models to scope post-fire success in restoring greater sage-grouse habitat. *Ecol Modell* **483**, 110396 (2023).

12. Prochazka, B. G. *et al.* Encounters with pinyon-juniper influence riskier movements in greater sage-grouse across the great basin. *Rangel Ecol Manag* **70**, 39–49 (2017).

13. Coates, P. S. *et al.* Pinyon and juniper encroachment into sagebrush ecosystems impacts distribution and survival of greater sage-grouse. *Rangel Ecol Manag* **70**, 25–38 (2017).

14. Dinkins, J. B., Lawson, K. J. & Beck, J. L. Influence of environmental change, harvest exposure, and human disturbance on population trends of greater sage-grouse. *PLoS One* **16**, 1–30 (2021).

15. Baruch-Mordo, S. *et al.* Saving sage-grouse from the trees: A proactive solution to reducing a key threat to a candidate species. *Biol Conserv* **167**, 233–241 (2013).

16. Olsen, A. C. *et al.* Reversing tree expansion in sagebrush steppe yields population-level benefit for imperiled grouse. *Ecosphere* **12**, e03551 (2021).

17. Donnelly, J. P. *et al.* Seasonal drought in North America's sagebrush biome structures dynamic mesic resources for sage-grouse. *Ecol Evol* **8**, 12492–12505 (2018).

18. Atamian, M. T., Sedinger, J. S., Heaton, J. S. & Blomberg, E. J. Landscape-level assessment of brood rearing habitat for greater sage-grouse in Nevada. *Journal of Wildlife Management* **74**, 1533–1543 (2010).

19. Aldridge, C. L. & Boyce, M. S. Linking occurrence and fitness to persistence: Habitat-based approach for endangered Greater Sage-Grouse. *Ecological Applications* **17**, 508–526 (2007).

20. Gibson, D., Blomberg, E. J., Atamian, M. T. & Sedinger, J. S. Weather, habitat composition, and female behavior interact to modify offspring survival in Greater Sage-Grouse: *Ecological Applications* **27**, 168–181 (2017).

21. Lundblad, C. G. *et al.* Sensitivity to weather drives Great Basin mesic resources and Greater Sage-Grouse productivity. *Ecol Indic* **142**, (2022).

22. Donnelly, J. P., Naugle, D. E., Hagen, C. A. & Maestas, J. D. Public lands and private waters: Scarce mesic resources structure land tenure and sage-grouse distributions. *Ecosphere* **7**, (2016).

23. Severson, J. P. *et al.* Moisture abundance and proximity mediate seasonal use of mesic areas and survival of greater sage-grouse broods. *Ecological Solutions and Evidence* **3**, (2022).

24. Casazza, M. L., Coates, P. S. & Overton, C. T. Linking habitat selection and brood success in Greater Sage-Grouse. in *Studies in Avian Biology: Ecology, conservation, and management of grouse* (eds. Sandercock, B. K., Martin, K. & Segelbacher, G.) vol. 39 151–167 (2011).

25. Thompson, K. M., Holloran, M. J., Slater, S. J., Kuipers, J. L. & Anderson, S. H. Early brood-rearing habitat use and productivity of greater sage-grouse in Wyoming. *West N Am Nat* **66**, 332–342 (2006).

26. O'Neil, S. T. *et al.* Wildfire and the ecological niche: Diminishing habitat suitability for an indicator species within semi-arid ecosystems. *Glob Chang Biol* **26**, 6296–6312 (2020).

27. Coates, P. S. *et al.* Wildfire, climate, and invasive grass interactions negatively impact an indicator species by reshaping sagebrush ecosystems. *Proc Natl Acad Sci U S A* **113**, 12745–12750 (2016).

28. Balch, J. K., Bradley, B. A., D'Antonio, C. M. & Gómez-Dans, J. Introduced annual grass increases regional fire activity across the arid western USA (1980-2009). *Glob Chang Biol* **19**, 173–183 (2013).

29. D'Antonio, C. M. & Vitousek, P. M. Biological invasion by exotic grasses, the grass/fire cycle, and global change. *Annu Rev Ecol Syst* **23**, 63–87 (1992).

30. Schroeder, M. A. & Robb, L. A. Fidelity of greater sage-grouse Centrocercus urophasianus to breeding areas in a fragmented landscape. in *Wildlife Biology* vol. 9 291–299 (Nordic Council for Wildlife Research, 2003).

31. Tyrrell, E. A. *et al.* Wildfire immediately reduces nest and adult survival of greater sage-grouse. *Sci Rep* **13**, 10970 (2023).

32. Anthony, C. R., Foster, L. J., Hagen, C. A. & Dugger, K. M. Acute and lagged fitness consequences for a sagebrush obligate in a post mega-wildfire landscape. *Ecol Evol* **12**, 1–12 (2022).

33. Xian, G. & Homer, C. Updating the 2001 National Land Cover Database impervious surface products to 2006 using Landsat imagery change detection methods. *Remote Sens Environ* **114**, 1676–1686 (2010).

34. Walker, B. L., Neubaum, M. A., Goforth, S. R. & Flenner, M. M. Quantifying habitat loss and modification from recent expansion of energy infrastructure in an isolated, peripheral greater sage-grouse population. *J Environ Manage* **255**, (2020).

35. Li, T. *et al.* Fragmentation of China's landscape by roads and urban areas. *Landsc Ecol* **25**, 839–853 (2010).

36. O'Neil, S. T. *et al.* Broad-scale occurrence of a subsidized avian predator: Reducing impacts of ravens on sage-grouse and other sensitive prey. *Journal of Applied Ecology* **55**, 2641–2652 (2018).

37. Manier, D. J. *et al.* Conservation Buffer Distance Estimates for Greater Sage-Grouse—a Review. *U.S. Geological Survey Open-File Report* 2014–1239, http://dx.doi.org/10.3133/ofr20141239. (2014).

38. Coates, P. S. *et al.* Geothermal energy production adversely affects a sensitive indicator species within sagebrush ecosystems in western North America. *Biol Conserv* **280**, 109889 (2023).

39. Walker, B. L., Naugle, D. E. & Doherty, K. E. Greater sage-grouse population response to energy development and habitat loss. *J Wildl Manage* **71**, 2644–2654 (2007).

Appendix S10

Post-hoc sensitivity analysis to investigate relative magnitude of precipitation/drought effects on sage-grouse vital rates (nest, brood, and adult survival)

We conducted a post-hoc analysis to evaluate and compare the relative magnitude of weather and climatic variables across the nest survival, brood survival, and adult survival life stages. We did this to aid interpretation of nuanced model responses to a suite of variables that were tested. Our post-hoc analysis focused on the change in cumulative survival within the life stage of interest, measured across the range of plausible values for each relevant predictor variable. Using the posterior distributions of parameter estimates from final model results, we applied the following method for each variable of interest and/or vital rate, defined as x with associated coefficient β_x:

1) We set all non-target covariate values (those other than x) to be held constant at their means
2) We then allowed only x to vary across a range of plausible values, defined as the 2.5^{th} – 97.5^{th} percentiles of the observed data
3) We drew 1000 samples from the posterior distributions of the intercept and β_x
4) For each sample, we predicted daily survival rate from the lower (2.5^{th} percentile) to the upper (97.5^{th} percentile) and exponentiated to the cumulative survival rate (*CSR*) at each end
5) We calculated percent change in cumulative survival; if survival declined across x, then percent decrease was calculated as (CSR_{start} - CSR_{final} / $|CSR_{start}|$) × 100; otherwise, percent increase was calculated as (CSR_{final} - CSR_{start} / $|CSR_{start}|$) × 100.

We summarized results for each modeled life stage:

Nest survival

The precipitation/drought variable of interest supported in our model of nest survival was concurrent vapor pressure deficit (*VPD*), a measure of aridity. The model indicated that nest survival declined with increasing *VPD*. The nesting period was defined as 38 days. *CSR* at low aridity was predicted to be 0.348 (95% CRI = 0.304–0.395) and at high aridity was predicted to be 0.244 (0.186–0.313), representing a **28.8% (-0.24%–50.8%) <u>decrease</u> in expected mean nest survival from low to high aridity**.

Brood survival

The precipitation/drought variables of interest supported in our model of brood survival were average minimum temperature (*TMIN*), previous fall standardized precipitation index (SPI_{fall}), and concurrent standardized precipitation index (*SPI*). Brood survival interaction effects were also supported for *TMIN* and $SPI_{fall,}$ with effects varying with brood age and day of season, respectively.

The model indicated that early brood survival increased with higher *TMIN* but that late brood survival decreased with higher *TMIN*. The early brood survival period was the first 21 days while the late brood survival period was the last 29 days. For early broods, *CSR* at low *TMIN* was predicted to be 0.684 (0.554–0.790) and at high *TMIN* was predicted to be 0.801 (0.709–0.872), representing a **18.4% (-5.7%–52.9%) <u>increase</u> in expected mean early brood survival from low to high *TMIN***. For late broods, *CSR* at low *TMIN* was predicted to be 0.835 (0.735–0.909) and at high *TMIN* was predicted to be 0.652 (0.472–0.801), representing a **22.1% (-2.3%–45.8%) <u>decrease</u> in expected mean late brood survival from low to high *TMIN***.

The model indicated that brood survival early in the season increased with SPI_{fall} but the effect decayed to negative late in the season. We assumed a 25-day period (half of total brood survival period). During the early season, *CSR* at low SPI_{fall} was predicted to be 0.521 (0.317–0.706) and at high SPI_{fall} was predicted to be 0.835 (0.750–0.902), representing a **67.9% (10.9%–179.7%) increase in expected mean early season brood survival from low to high SPI_{fall}.** In contrast, during the late season, *CSR* at low SPI_{fall} was predicted to be 0.852 (0.734–0.927) and at high SPI_{fall} was predicted to be 0.651 (0.482–0.789), representing a **23.1% (-0.49%–46.07%) decrease in expected mean late season brood survival from low to high SPI_{fall}.**

The model indicated that brood survival declined in response to concurrent *SPI*, an effect that did not vary depending on season or brood age. The total brood period was 50 days. *CSR* at low *SPI* was predicted to be 0.640 (0.560–0.715) and at high *SPI* was predicted to be 0.468 (0.370–0.560), a **26.6% (7.61%–43.76%) decrease in expected mean brood survival from low to high concurrent *SPI***.

Adult survival

The precipitation/drought variables of interest supported in our model of adult survival were concurrent VPD, previous fall standardized precipitation evapotranspiration index ($SPEI_{fall}$), and spring snow-water equivalent (*SWE*). Interaction effects were also supported for $SPEI_{fall}$, with effects varying with day of season.

The model indicated that adult survival increased in response to concurrent *VPD*, an effect that did not vary depending on day of season. The total reproductive period for adults was 167 days. *CSR* at low *VPD* was predicted to be 0.682 (0.582–0.768) and at high *VPD* was predicted to be 0.845 (0.768–0.900), a **24.6% (4.49%–49.88%) increase in expected mean survival from low to high concurrent *VPD***.

The model indicated that adult survival early in the season increased with $SPEI_{fall}$ with a slight decay in the effect later in the season. During the early season, *CSR* at low $SPEI_{fall}$ was predicted to be 0.615 (0.443–0.747) and at high $SPEI_{fall}$ was predicted to be 0.794 (0.699–0.861), representing a **31.02% (5.58%–77.68%) increase in expected survival from low to high $SPEI_{fall}$.** During the late season, *CSR* at low $SPEI_{fall}$ was predicted to be 0.711 (0.571–0.822) and at high $SPEI_{fall}$ was predicted to be 0.818 (0.731–0.882), representing a **15.96% (-4.40%–44.27%) increase in expected mean survival from low to high SPI_{fall}.**

The model indicated that adult survival decreased in response to spring *SWE*, an effect that did not vary depending on day of season. *CSR* at low *SWE* was predicted to be 0.766 (0.714–0.812) and at high *SWE* was predicted to be 0.697 (0.609–0.768), a **9.2% (1.71%–17.59%) decrease in expected mean survival from low to high spring *SWE***.
